# The effect of random virus failure following cell entry on infection outcome and the success of antiviral therapy

**DOI:** 10.1038/s41598-023-44180-w

**Published:** 2023-10-11

**Authors:** Christian Quirouette, Daniel Cresta, Jizhou Li, Kathleen P. Wilkie, Haozhao Liang, Catherine A. A. Beauchemin

**Affiliations:** 1 Department of Physics, Toronto Metropolitan University, Toronto, Canada; 2 Interdisciplinary Theoretical and Mathematical Sciences (iTHEMS), RIKEN, Wako, Japan; 3 Department of Mathematics, Toronto Metropolitan University, Toronto, Canada; 4 Nishina Center for Accelerator-Based Science (RNC), RIKEN, Wako, Japan; 5 Present Address: Department of Physics, University of Tokyo, Tokyo, Japan

**Keywords:** Virology, Applied mathematics

## Abstract

A virus infection can be initiated with very few or even a single infectious virion, and as such can become extinct, i.e. stochastically fail to take hold or spread significantly. There are many ways that a fully competent infectious virion, having successfully entered a cell, can fail to cause a productive infection, i.e. one that yields infectious virus progeny. Though many stochastic models (SMs) have been developed and used to estimate a virus infection’s establishment probability, these typically neglect infection failure post virus entry. The SM presented herein introduces parameter $\gamma \in (0,1]$ which corresponds to the probability that a virion’s entry into a cell will result in a productive cell infection. We derive an expression for the likelihood of infection establishment in this new SM, and find that prophylactic therapy with an antiviral reducing $\gamma$ is at least as good or better at decreasing the establishment probability, compared to antivirals reducing the rates of virus production or virus entry into cells, irrespective of the SM parameters. We investigate the difference in the fraction of cells consumed by so-called extinct versus established virus infections, and find that this distinction becomes biologically meaningless as the probability of establishment approaches zero. We explain why the release of virions continuously over an infectious cell’s lifespan, rather than as a single burst at the end of the cell’s lifespan, does not result in an increased risk of infection extinction. We show, instead, that the number of virus released, not the timing of the release, affects infection establishment and associated critical antiviral efficacy.

## Introduction

Typically, mathematical models describing the course of a virus infection within a host (in vivo) or a cell culture (in vitro) express the number of cells and infectious virions (virus particles) as real positive numbers (continuous), and infection events as deterministic, e.g. one virion infects 0.2 cell. By nature, however, the number of cells and virions are whole numbers (discrete) and infection events are stochastic, e.g. one virion will infect one cell 20% of the time. When dealing with large numbers of infectious virions and cells, stochastic fluctuations can become negligible, and the continuous, deterministic, mean-field approach can provide an accurate representation of the kinetics of interest. Yet the small number regime arises commonly. For example, antiviral therapy can reduce the effective number of infection-capable virions to near or below unity. In such cases, random fluctuations could have an important effect on the time course and outcome of an infection.

**Figure 1:**
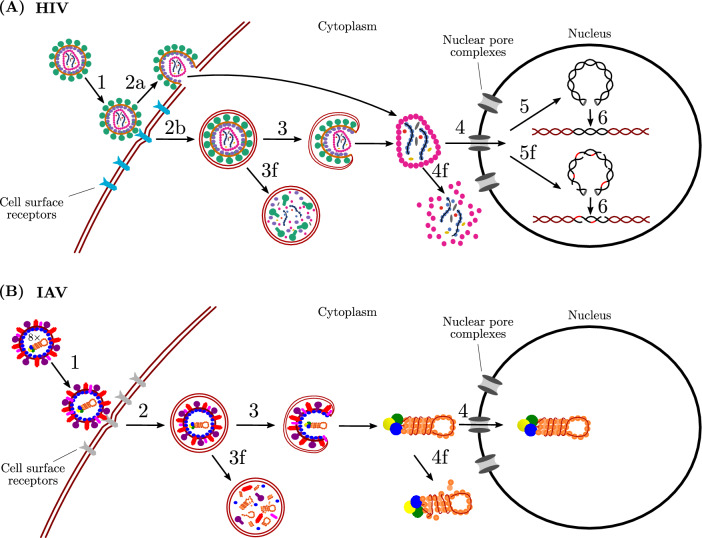
Early steps of HIV and IAV replication and possible routes of abortive cell infections. (**A**) An HIV virion attaches to the cell surface (Step 1), and can enter by direct fusion of the virus membrane with the cell surface (Step 2a) or be endocytosed (Step 2b). Endosomal fusion can be successful and the virus genomic material is released into the cell (Step 3) or fails and the virus-containing endosome is eventually degraded (Step 3f). Following successful entry, the virus genomic material can be imported into the nucleus (Step 4) or fail to do so and eventually be subject to degradation (Step 4f). Reverse transcription, i.e. viral RNA is converted to viral DNA, either proceeds successfully (Step 5) or fatal errors are introduced into the viral DNA during reverse transcription (Step 5f). Once in the nucleus, viral DNA is integrated into the cell DNA (Step 6) and can later be used for virus replication. (**B**) An IAV virion attaches to the cell surface (Step 1). The virion then enters by endocytosis (Step 2). Next, endosomal fusion can be successful (Step 3) or fail (Step 3f). If fusion is successful, the released viral ribonucleoproteins (vRNPs) containing the viral RNA segments can either all be imported into the nucleus (Step 4) or some might fail to enter the nucleus and be degraded (Step 4f). Once vRNPs are inside the nucleus, virus replication is then initiated. For both viruses, as the number of copies of viral RNA and proteins grow during replication, it becomes increasingly unlikely that the cell infection will fail to produce any infectious progeny.

Several stochastic models (SMs) of virus infection kinetics have been proposed to date, e.g.^1–7^. Notably, Heldt et al.^4^ predicted with an intracellular SM that most cells inoculated with a single infectious virion would result in a non-productive cell infection. But extracellular SMs typically do not account for cell infection failure post virus entry. When it has been included only some lethal replication errors were represented, e.g. lethal reverse transcription errors only^6^. In fact, there are many ways that infectious virions post cell entry can fail to cause a cell infection that will produce fully infectious progeny. Fig. 1 illustrates early steps of human immunodeficiency virus (HIV) and influenza A virus (IAV) replication and associated opportunities for abortive cell infections.

For HIV, entry begins with successful binding to the cell surface, followed by fusion of the virus membrane with the cell surface. Yet sometimes virions can be taken in via the endocytic route^8^. In this case, virions can fail to fuse with late endosomes and eventually be degraded^9^. Or, if there is successful entry, virions can still be degraded in the cytosol^10^. The virus can also fail to be imported into the nucleus^11,12^. In addition, fatal mutations can be acquired during reverse transcription^13^, when viral RNA is transcribed into complementary DNA (cDNA) which is used to make typically a single copy of viral DNA for integration. Finally, failure may also be the result of host cell mechanisms that interfere with early replication steps^14,15^.

For IAV, entry begins with successful binding to the cell surface, followed by internalization by endocytosis. Failure to undergo fusion with late endosomes can then occur^16,17^. If uncoating happens, released viral ribonucleoproteins (vRNPs) containing the viral RNA segments could be subject to RNA degradation in the cytosol^18^ or fail to enter the nucleus^17,19^. Degradation of one or more genome segments, following nuclear import and prior to transcription could also occur^4^. Since each genome segment encodes at least one viral protein, degradation of only one of them means failure to cause a productive cell infection. There is also a multitude of host cell mechanisms that can impair early steps of virus replication^20–22^ and these may be yet more potential sources for failure.

These are just some of the ways in which otherwise fully physically infectious virions could, through random chance, fail to complete a key step following cell entry. In addition to such stochastic occurrences in fully functional virions, a number of entry capable virions could have physical defects that prevents them from completing one or more key replication steps, leaving them physically unable to cause a productive infection. As such, failure of productive cell infection post virus entry is a combination of both the stochastic post-entry failure of otherwise fully infectious virions, and the inevitable failure of replication defective, entry capable virions.

A common, important application of SMs is to estimate the extinction probability of an infection: the likelihood that the infection will fail to take hold or spread significantly. It typically depends on both the replication capabilities of the virus (i.e. infection parameters) and the initial number of infectious virions or cells. The extinction probability is an important quantity to derive as it can, for example, be used to evaluate the probability of success of antiviral therapy^3,23^. In particular, Czuppon et al.^23^ compared the ability of prophylactic antivirals acting either on the rate of virus entry into cell or the rate of virus production, to reduce the establishment probability, or $1-$(extinction probability), of severe acute respiratory syndrome coronavirus 2 (SARS-CoV-2) infection in vivo. However, the effect of certain classes of antivirals, such as endosomal fusion inhibitors, would be better represented as reducing the probability that, having successfully entered a cell, a virion will go on to cause the productive infection of that cell. For IAV, it would be a more appropriate way to represent the mode of action of adamantanes, such as amantadine and rimantadine which block the M2 ion channel necessary for successful fusion of IAV with the endosome^24,25^. For SARS-CoV-2, it has been suggested that cathepsin L inhibitors can reduce the probability of successful endosomal fusion^26^. Failure to undergo endosomal fusion would lead to loss of the infectious virion, but would not result in a productive cell infection.

Although the extinction probability is often an important consideration in comparing prophylactic antivirals, what is never discussed is the number or fraction of cells that are actually consumed by infections that are said to have gone “extinct” or to have “established”. It is possible that antivirals with different modes of action, even with the same extinction probability, could result in a very different fraction of cells consumed by so-called established or extinct infections. This would have important implications for how one should interpret the probability of success of a particular antiviral therapy.

In addition, nearly all SMs, including past works that used the extinction probability to evaluate the probability of success of antiviral therapy^3,23^, assume the duration of the infectious phase, the period during which infected cells are producing and releasing virus progeny, to be exponentially distributed. Careful pairing of mathematical models and experimental measurements has established that the duration of the infectious phase in vitro for cells infected with IAV^27^, simian HIV^28^, or Ebola virus^29^, follows a log-normal or normal-like distribution, and has clearly rejected the probability of an exponentially distributed infectious phase duration. Yan et al.^5^ estimated the extinction probability for a SM of IAV infection in vivo that allowed the lifespan of infectious cells to follow an Erlang distribution which, via its shape parameter (*k*), can capture exponential ($k=1$), log-normal-like ($k\sim [1,6]$), normal-like ($k>10$), and even Dirac delta-like ($k\rightarrow \infty$) distributions. Yan et al.^5^ have shown that increasing the shape parameter (*k*) from exponential to log-normal leads to a decrease in the extinction probability given an infection initiated with a number of infectious virions. Hence, the distribution of the infectious phase duration is also expected to affect the likelihood that an infection will become established under antiviral therapy.

In this work, we construct a SM for virus infection through the physical consideration of key infection steps, rather than through a systematic, direct mathematical conversion of our mean-field model. Our SM explicitly represents the probability that a virion, after having successfully entered a cell, will fail to result in the productive infection of that cell. The SM is first used to estimate the extinction probability of an infection. Extending work by Czuppon et al.^23^, we show that prophylactic therapy with an antiviral that blocks productive cell infection *after* virus entry, is better at reducing the establishment probability, than one reducing either the virus production rate or the rate of virus entry into cells. In addition, we investigate the difference in the fraction of cells consumed by so-called extinct versus established infections, and re-visit the comparison of antivirals through this new lens. Finally, we demonstrate how the antiviral efficacy required to achieve a desired infection extinction probability critically depends on the assumed distribution of the infectious phase duration.

## Results

### The mathematical models

**Figure 2:**
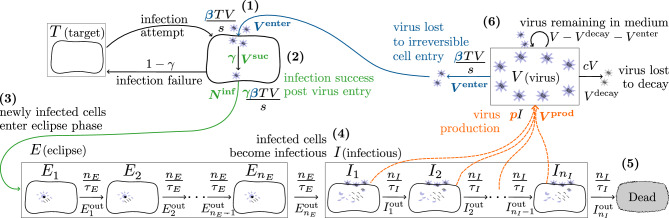
Representation of the mathematical models. (1) As uninfected, susceptible target cells (*T*) interact with infectious virions (*V*) over time, some portion of the latter ($V^\text {enter}$) irreversibly enter some of the available target cells, at rate $\beta T V/s$. (2) Out of these, a fraction $\gamma \in (0,1]$ will cause the successful infection of $N^\text {inf}$ target cells, while infection failure of the remaining fraction $(1-\gamma )$ leaves cells in their uninfected state. (3) A newly infected cell will first enter the eclipse phase, and (4) then transition into the infectious phase during which it produces infectious virions at an average rate *p*, before (5) ultimately ceasing virus production and possibly undergoing apoptosis. (6) At each time step in the SM, a random number of newly produced infectious virions ($V^\text {prod}$) are released into the medium or organ compartment of volume $s$, increasing the infectious virus concentration already present, while $V^\text {decay}$ infectious virions will lose infectivity, and another $V^\text {enter}$ will be lost to irreversible cell entry. Terms like $\beta T V/s$, $n_E/\tau _E$, $n_I/\tau _I$, *pI*, and *cV* represent the rates at which events take place in the MFM, whereas terms like $V^\text {enter}$, $E_i^\text {out}$, $I_j^\text {out}$, $V^\text {prod}$, and $V^\text {decay}$ correspond to the random number of such events over one time step of the SM (see Table 1).

The mean-field mathematical model (MFM), its stochastic counterpart, and associated infection parameters are illustrated in Fig. 2. Mathematically, the MFM and SM are given by

1 $$\begin{aligned} \frac{\textrm{d}T}{\textrm{d}t}&= -\gamma \beta TV/s&T^{t+1}&= T^t-N^{\text {inf}} \nonumber \\ \frac{\textrm{d}E_1}{\textrm{d}t}&= \gamma \beta TV/s- \frac{n_E}{\tau _E} E_1&E_1^{t+1}&= E_1^t + N^{\text {inf}} - E_1^{\text {out}} \nonumber \\ \frac{\textrm{d}E_i}{\textrm{d}t}&= \frac{n_E}{\tau _E}E_{i-1} - \frac{n_E}{\tau _E}E_i&E_i^{t+1}&= E_i^t + E_{i-1}^{\text {out}} - E_i^{\text {out}} \qquad i=2,3,...,n_E \nonumber \\ \frac{\textrm{d}I_1}{\textrm{d}t}&= \frac{n_E}{\tau _E}E_{n_E}-\frac{n_I}{\tau _I}I_1&I_1^{t+1}&= I_1^t + E_{n_E}^{\text {out}} - I_1^{\text {out}} \nonumber \\ \frac{\textrm{d}I_j}{\textrm{d}t}&= \frac{n_I}{\tau _I}I_{j-1}-\frac{n_I}{\tau _I}I_j&I_j^{t+1}&= I_j^t + I_{j-1}^{\text {out}} - I_j^{\text {out}} \qquad j=2,3,...,n_I \nonumber \\ \frac{\textrm{d}V}{\textrm{d}t}&= p\sum _{j=1}^{n_I}I_j-cV-\beta _{} TV/s&V^{t+1}&= V^t + V^\text {prod} - V^\text {decay} - V^\text {enter} \end{aligned}$$

Initially, all cells are uninfected, susceptible target cells, *T*, i.e. $T(t=0) = N_\text {cells}$, which are exposed to an initial dose of $V(t=0)=V_0$ infectious virions. As target cells, *T*, encounter infectious virions, *V*, in the supernatant of a cell culture, a tissue, or an organ compartment of volume $s$, some become infected ($T\rightarrow E_1$). The rate of successful cell infections by infectious virions is $\gamma \beta V/s$, which depends on the concentration of infectious virions $V/s$. When a cell becomes infected, it enters ($T\rightarrow E_1$) and traverses ($E_1\rightarrow E_2\rightarrow \ldots \rightarrow E_{n_E}$) the $n_E$ compartments of the eclipse phase, during which it is infected but not yet producing infectious virions. The infected cell then enters ($E_{n_E}\rightarrow I_1$) and traverses ($I_1\rightarrow I_2\rightarrow \ldots \rightarrow I_{n_I}$) the infectious phase, during which it produces infectious virions at constant rate *p*. When an infected cell leaves the last compartment ($I_{n_I}$), it ceases virus production and thus ceases to contribute to the infection kinetics, and possibly undergoes apoptosis. The exponentially distributed durations of the $n_E$ eclipse (or $n_I$ infectious) phase compartments together yield an Erlang distributed total duration for the eclipse (or infectious) phase of mean duration $\tau _E$ (or $\tau _I$), and standard deviation $\tau _E/\sqrt{n_E}$ (or $\tau _I/\sqrt{n_I}$), where $n_E$ (or $n_I$) corresponds to the shape parameter of the Erlang distribution. These compartments are not meant to correspond to particular biological states. Rather, they offer a mathematically and computationally expeditious way (compared to delayed, partial or integro-differential equations^27,30^) to implement biologically realistic durations for the time spent by infected cells in the eclipse and infectious phases (e.g., normal and lognormal distributions^28,29,31^).

Infectious virions (*V*) are produced at a constant rate of *p* per infectious cell, and are lost either through loss of infectivity at rate *c*, or irreversible commitment to entry into susceptible cells at rate $\beta T/s$. Here, $\beta T/s$ is not meant to represent the rate of virus loss due to cell attachment because attachment is a reversible process: virions can still either detach and re-join the pool of virions in the extracellular space or irreversibly enter the cell. Instead, given the ongoing kinetics of virus attachment/detachment, $\beta T/s$ represents the net rate at which virions irreversibly commit to cell entry, i.e. pass the point of no return from which they cannot re-enter the extracellular space. For brevity, we will refer to $\beta$ hereafter simply as the rate of virus loss due to cell entry.

This MFM is similar to that widely validated and applied to analyze and predict the course of in vitro infections with IAV^32–34^, simian HIV^28^, Ebola virus^29^, RSV^35^ and rotavirus^36^. It differs from our previous MFM by explicitly accounting for the loss of infectious virions due to cell entry (term $-\beta TV/s$ in the $\textrm{d}V/\textrm{d}t$ equation), previously considered by others^2,5,37,38^. This loss of infectious virions due to cell entry causes a corresponding loss of uninfected target cells becoming infected ($T\rightarrow E_1$) at rate $\gamma \beta TV/s$. Parameter $\gamma$ therefore has units of cell per infectious virion (IV), where $\gamma \in {(0,1]}\,{\textrm{cell}/\text {IV}}$, and corresponds to the average fraction of infectious virion entries into a cell that results in the successful, productive infection of that cell ($T\rightarrow E_1$). This additional parameter $\gamma$ distinguishes our work from past work^2,5,37,38^ where it has been assumed that the loss of one infectious virion due to cell entry always inevitably leads to the successful infection of one cell, i.e. $\gamma = 1$ infected cell per infectious virion entry. We use this assumption as our base parameter value for $\gamma$ throughout the work presented herein.

Biologically, $\gamma$ accounts for several different causes of infection failure after a virion has irreversibly committed to entry into a cell, e.g. Fig. 1. It can represent semi-infectious virions that are entry-competent, but are defective in their ability to complete another downstream step, e.g. are missing one or more viral genome segment or have deleterious genetic mutations. It can also represent fully infectious virions that are not defective but rather, through random chance, fail to achieve a key step they could have functionally achieved, e.g. 50% of influenza A virions failing to fuse with endosome membrane following cell entry^16,39^. Herein it is assumed that when an infectious virion enters a cell, the cell will either be successfully infected ($T\rightarrow E_1$) with probability $\gamma$, or remain uninfected (*T*) with probability $1-\gamma$. Failed infections are assumed to have no significant effect, leaving the uninfected cell exactly as they found it in state *T*. Upon infection, we assume the cell becomes instantaneously unavailable for co-infection, i.e. virions are lost through entry into target cells only, and cannot enter infected cells. As such, we do not attempt to represent the case of co-infection of a cell by semi-infectious, segmented RNA virions, each with an incomplete set of functional genome segments, which together have a complete set and are thus able to cause a productive cell infection^40,41^. We also do not include defective interfering particle production^42^. Since our work focuses on infections starting from very few infectious virions, where randomness plays a meaningful role, the likelihood of cell co-infection and these associated effects should be negligible.

The SM is largely analogous to the MFM, where SM variables, e.g. $T^t$ the number of target cells at time *t*, each denoted with a superscript *t*, are whole numbers. The remaining terms, corresponding to changes in these variables, are random whole numbers drawn at each time step from distributions thought to best represent the corresponding underlying biological process (see Methods). Table 1 summarizes how each random variable is generated.

**Table 1:** Random variables of the stochastic model.

Random variable	Random number generator
$E_i^\text {out}$	$\text {Binomial}(n=E_i^t,\, p_E=\Delta t\cdot n_E/\tau _E)$ where $i=1,2,...,n_E$
$I_j^\text {out}$	$\text {Binomial}(n=I_j^t,\, p_I=\Delta t\cdot n_I/\tau _I)$ where $j=1,2,...,n_I$
$V^\text {prod}$	$\text {Poisson}(\lambda =\Delta t\cdot p \sum _{j=1}^{n_I} I_j^t)$
$V^\text {decay},V^\text {enter},\text {otherwise}$	$\text {Trinomial}(n=V^t,\, p_1=\Delta t\cdot c,\, p_2=\Delta t\cdot \beta T^t/s,\, p_3=1-p_1-p_2)$
$N^\text {inf}$	$|\{x_i | x_i \in {\mathcal {U}}\{a=1,\, b=T^t\}, 1 \le i \le V^\text {suc}\}|^\dagger$
	where $V^\text {suc} = \text {Binomial}(n=V^{\text {enter}},\,p_V=\gamma )$

$^\dagger$ $N^\text {inf}$ is equal to the cardinality, i.e. the number of unique elements, in the set of $V^\text {suc}$ random numbers drawn from the discrete uniform distribution over the interval $[1,T^t]$.

### Important biological quantities

Following Pearson et al.^2^, let us define ${\mathcal {P}}_{V \rightarrow \, \text {Extinction}}$ and ${\mathcal {P}}_{I \rightarrow \, \text {Extinction}}$ as the probability of infection extinction given an infection initiated with either only one infectious virion or only one infectious cell, respectively. We now rewrite the derivation from Pearson et al.^2^ for these quantities, in the context of our SM and its associated parameter notation. Unless otherwise stated, equivalent equations can be found in Pearson et al.^2^.

The extinction probability given any initial number $V_0$ of infectious virions and $I_0$ of infectious cells is $\left( {\mathcal {P}}_{V \rightarrow \, \text {Extinction}}\right) ^{V_0} \cdot \left( {\mathcal {P}}_{I \rightarrow \, \text {Extinction}}\right) ^{I_0}$. If initially there is only one infectious virion, the infection can fail to spread if either that initial infectious virion fails to cause a successful cell infection with probability $1-{\mathcal {P}}_{V \rightarrow \, I }$, or if it does cause a successful cell infection with probability ${\mathcal {P}}_{V \rightarrow \, I }$ but that cell infection subsequently leads to extinction with probability ${\mathcal {P}}_{I \rightarrow \, \text {Extinction}}$. We can therefore write

2 $$\begin{aligned} {\mathcal {P}}_{V \rightarrow \, \text {Extinction}}= (1-{\mathcal {P}}_{V \rightarrow \, I })+{\mathcal {P}}_{V \rightarrow \, I }\cdot {\mathcal {P}}_{I \rightarrow \, \text {Extinction}}\ . \end{aligned}$$

If initially there is only one infectious cell, the cell will produce *m* infectious virions over its lifespan with probability ${\mathcal {P}}_{I \rightarrow \, m \, V}$. Each one of these produced infectious virions can be treated as an independent infection event such that,

3 $$\begin{aligned} {\mathcal {P}}_{I \rightarrow \, \text {Extinction}}= \sum _{m=0}^\infty {\mathcal {P}}_{I \rightarrow \, m \, V}\cdot \left( {\mathcal {P}}_{V \rightarrow \, \text {Extinction}}\right) ^m \ . \end{aligned}$$

Taken together, the extinction probability given an infection initiated with only one infectious virion, ${\mathcal {P}}_{V \rightarrow \, \text {Extinction}}$, yields the implicit relation

4 $$\begin{aligned} {\mathcal {P}}_{V \rightarrow \, \text {Extinction}}= (1-{\mathcal {P}}_{V \rightarrow \, I })+{\mathcal {P}}_{V \rightarrow \, I }\cdot \sum _{m=0}^\infty {\mathcal {P}}_{I \rightarrow \, m \, V}\cdot \left( {\mathcal {P}}_{V \rightarrow \, \text {Extinction}}\right) ^m \ . \end{aligned}$$

The probability that an infectious virion is successful at causing a productive cell infection, ${\mathcal {P}}_{V \rightarrow \, I }$, can be expressed as the ratio between the rate of successful cell infection per infectious virion $\gamma \beta N_\text {cells}/s$ and the rate of virion loss through loss of infectivity plus cell entry, $c+\beta N_\text {cells}/s$ (see Methods for derivation), namely,

5 $$\begin{aligned} {\mathcal {P}}_{V \rightarrow \, I }= \frac{\gamma \beta N_\text {cells}/s}{c+\beta N_\text {cells}/s} = \frac{\gamma }{1+c/(\beta N_\text {cells}/s)} \ . \end{aligned}$$

This expression reduces to that presented in Pearson et al.^2^ when one assumes that the loss of one infectious virion due to cell entry always results in the infection of one cell, i.e. $\gamma ={1}\,{\textrm{cell}/\text {IV}}$.

The probability that an infectious cell produces *m* infectious virions over its lifespan, ${\mathcal {P}}_{I \rightarrow \, m \, V}$, can be expressed as the marginal probability distribution of the probability that an infectious cell produces *m* infectious virions given a lifespan of length *t*, $\text {Poisson}(m|\lambda =pt)$, and the probability that the lifespan is of length *t*, $\text {Erlang}(t|k=n_I,\lambda =n_I/\tau _I)$. Therefore,

6 $$\begin{aligned} {\mathcal {P}}_{I \rightarrow \, m \, V}&= \int _0^\infty \text {Poisson}(m|\lambda =pt) \cdot \text {Erlang}(t|k=n_I,\, \lambda =n_I/\tau _I)\ \text {d}t \nonumber \\&= \frac{(m+r-1)!}{m!(r-1)!} (1-p_B)^r (p_B)^m = \text {NB}(m|r=n_I,\, p_B={\mathcal {B}}/(n_I+{\mathcal {B}})) \end{aligned}$$

where NB stands for the negative binomial (or Pascal) distribution and describes the probability that *m* successes will have occurred by the time one has observed $r=n_I$ failures from a series of independent Bernoulli trials with a probability of success $p_B$ (see Methods for derivation). The mean of the distribution corresponds to the average burst size, ${\mathcal {B}} = p \tau _I$, i.e. the average number of infectious virions produced by a productively infected cell over its lifespan, with *p* the rate of infectious virion production per cell and $\tau _I$ the average infectious cell lifespan. This expression for ${\mathcal {P}}_{I \rightarrow \, m \, V}$ is novel, and it does not have a corresponding equation in Pearson et al.^2^.

Combining the mean number of infectious virions produced by a productively infected cell, ${\mathcal {B}}$, and the probability that each of them causes the infection of a cell, ${\mathcal {P}}_{V \rightarrow \, I }$ (Eq. 5), we obtain the average basic reproductive number ($R_0$), defined as the average number of productive secondary infections caused by an infected cell over its lifespan when placed within a fully susceptible and uninfected cell population,

7 $$\begin{aligned} R_0 = {\mathcal {B}} \cdot {\mathcal {P}}_{V \rightarrow \, I }= p \tau _I \cdot \frac{\gamma \beta N_\text {cells}/s}{c+\beta N_\text {cells}/s} = p \tau _I \cdot \frac{\gamma }{1+c/(\beta N_\text {cells}/s)} \ . \end{aligned}$$

Substituting Eqs. (5) and (6) into Eq. (4) results in the following expression for ${\mathcal {P}}_{V \rightarrow \, \text {Extinction}}$

8 $${\mathcal{P}}_{{V \to {\text{Extinction}}}} = \left[ {1 - \frac{\gamma }{{1 + c/(\beta N_{{{\text{cells}}}} /s)}}} \right] + \frac{\gamma }{{1 + c/(\beta N_{{{\text{cells}}}} /s)}}\left[ {\frac{{{\mathcal{B}}(1 - {\mathcal{P}}_{{V \to {\kern 1pt} {\text{Extinction}}}} )}}{{n_{I} }} + 1} \right]^{{ - n_{I} }}$$

While Eq. (8) does not lead to an analytical solution for ${\mathcal {P}}_{V \rightarrow \, \text {Extinction}}$, a solution can easily be obtained numerically (see Methods). The probability of infection establishment given an infection initiated with only one infectious virion, ${\mathcal {P}}_{V \rightarrow \, \text {Establishment}}$, is simply $1-{\mathcal {P}}_{V \rightarrow \, \text {Extinction}}$.

### Effectiveness of antivirals to reduce an infection’s establishment probability

Prophylactic antiviral therapies are often characterized and compared in terms of their ability to reduce an infection’s establishment probability. This is because for natural infections in a host, the initial virus inoculum can be sufficiently small that prophylactic antiviral therapy can prevent infection, i.e. induce infection extinction.

**Figure 3:**
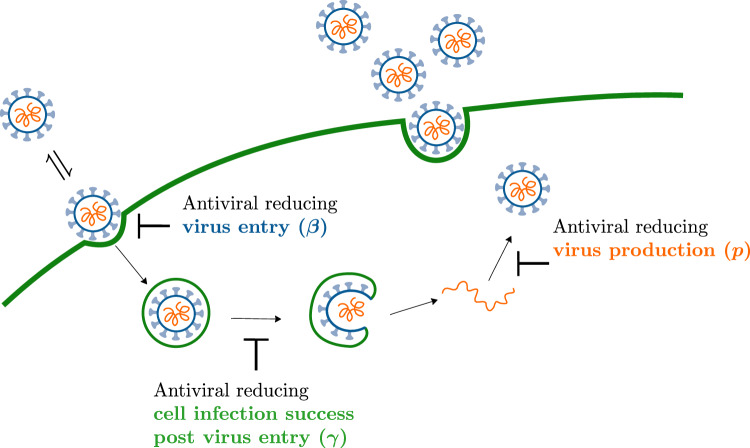
Antiviral modes of action. Antiviral reducing (left) the virus entry rate $\beta$, (centre) the probability of a successful cell infection post virus entry $\gamma$, or (right) the virus production rate *p*. If an antiviral reducing $\beta$ prevents an infectious virion from entering a cell, the virion could try again later on and succeed. Whereas, if an antiviral reducing $\gamma$ prevents an infectious virion from completing a successful cell infection post cell entry, the virion would be removed from the overall infection. Also, an antiviral reducing *p* would reduce virus production rather than totally block it, as would be the case if an antiviral reducing $\gamma$ prevents a productive cell infection. These differences between the modes of actions previously studied (reducing $\beta$ or *p*) and newly considered herein (reducing $\gamma$) highlight why the latter might prove advantageous.

Recently, Czuppon et al.^23^ used a SM to evaluate the effectiveness of prophylactic treatment with antivirals reducing the rate of virus entry into cells sometimes called the virus infectivity rate, herein $\beta$, or the virus production rate, *p*, to reduce the establishment probability for a SARS-CoV-2 infection in vivo initiated by a small number of infectious virions. But an endosomal fusion inhibitor (e.g. cathepsin L inhibitors for SARS-CoV-2^26^) would not affect the rate of removal of infectious virions from the medium (i.e. it leaves $\beta$ unaffected), but by enhancing fusion failure post cell entry, it would prevent the cell from progressing to a productively infectious state, hence blocking 100% of virion progeny production. Using our SM, which can explicitly represent productive infection failure post virus entry via parameter $\gamma$, we extend Czuppon’s investigation to evaluate how such an antiviral would compare against those reducing $\beta$ or *p*. Fig. 3 illustrates the different antiviral modes of action considered herein.

**Figure 4:**
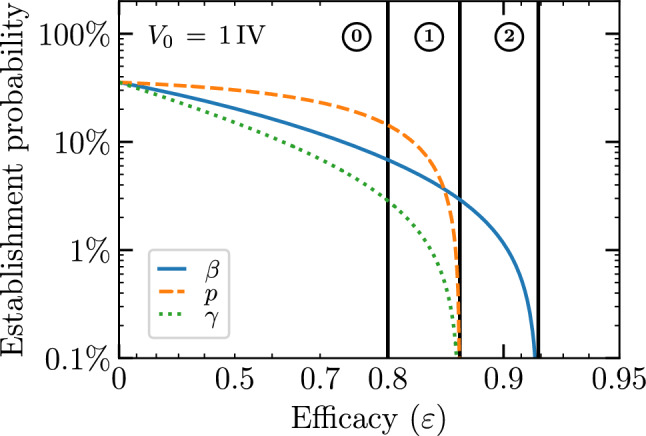
Effect of different antivirals on the probability of infection establishment. The establishment probability given an infection initiated with only one infectious virion as a function of efficacy ($\varepsilon$) for antivirals acting either to reduce the virus entry rate, $\beta \rightarrow (1-\varepsilon )\beta$, the virus production rate, $p \rightarrow (1-\varepsilon )p$, or the probability of a successful cell infection post virus entry, $\gamma \rightarrow (1-\varepsilon )\gamma$. The line labelled $\textcircled {0}$ indicates a value of lower efficacy ($\varepsilon = 0.8$), at line $\textcircled {1}$ the establishment probability goes to zero ($R_0=1$) for an antiviral reducing *p* or $\gamma$ ($\varepsilon \approx 0.87$), and at $\textcircled {2}$ it goes to zero for an antiviral reducing $\beta$ ($\varepsilon \approx 0.92$). The infection parameters are from Czuppon et al.^23^ (see Methods).

Figure 4 shows the establishment probability given an infection initiated with only one infectious virion as a function of the efficacy ($\varepsilon$) for antivirals reducing $\beta$, *p* or $\gamma$. As antiviral efficacy $\varepsilon$ increases, the establishment probability decreases at first slowly and then abruptly as it approaches zero (as the basic reproductive number, $R_0$, approaches one). As others before us^3,23^, we assume that these antivirals have negligible cytotoxic effects, i.e. do not cause cell damage or death, over the range of concentrations (efficacies) explored, and that all antivirals can achieve all efficacies. In reality, antivirals with different mechanisms of action would likely differ in their cytotoxicity profile, and in the maximum efficacy they can achieve following the $E_\text {max}$ model^43^, which relates drug concentration to efficacy. The parameter values used to generate this figure and all other figures herein are provided in the Methods. They were taken from Czuppon et al.^23^ for SARS-CoV-2 infections in patients, and in particular assume an exponentially distributed duration for the infectious phase ($n_I=1$). For $n_I=1$, the expression for the establishment probability in Eq. (8) reduces to

9 $$\begin{aligned} {\mathcal {P}}_{V \rightarrow \, \text {Establishment}}= {\mathcal {P}}_{V \rightarrow \, I }- \frac{1}{{\mathcal {B}}} = \frac{R_0}{{\mathcal {B}}} - \frac{1}{{\mathcal {B}}} = \frac{\gamma }{1+c/(\beta N_\text {cells}/s)} - \frac{1}{p \tau _I} \end{aligned}$$

where ${\mathcal {B}} =p\tau _I$ is the burst size (see Methods). Since $\beta$ and *c* appear as a ratio, $c/(\beta N_\text {cells}/s)$, in the expression for the establishment probability (Eq. (9)), an antiviral reducing $\beta$ and increasing *c* would have the same effect on the establishment probability as one only reducing $\beta$ with greater efficacy. This is particularly interesting because many antivirals that reduce the ability of infectious virions to enter cells (reduce $\beta$), such as monoclonal antibodies, often target the virions directly by binding to and disabling their surface protein, thus also enhancing the rate at which virions lose infectivity (increasing *c*).

At relatively low efficacy, e.g. $\varepsilon = 0.8$ (see line $\textcircled {0}$ in Fig. 4), for the parameters chosen, an antiviral reducing $\gamma$ is more effective than one reducing $\beta$, which is more effective than one reducing *p* at reducing the establishment probability. The infection parameters used by Czuppon et al.^23^ correspond to a sufficiently large burst size (${\mathcal {B}} =p\tau _I={18.8}\,{\mathrm{IV/cell}}$, see Methods) such that ${\mathcal {P}}_{V \rightarrow \, \text {Establishment}}\approx {\mathcal {P}}_{V \rightarrow \, I }$ ($=0.409$). As such, the bottleneck to infection establishment is the probability that the single initial infectious virion causes a productive cell infection, ${\mathcal {P}}_{V \rightarrow \, I }$, which depends on $\beta$ and $\gamma$, but not *p*. While an antiviral reducing $\beta$ reduces both the numerator and denominator of ${\mathcal {P}}_{V \rightarrow \, I }$, one reducing $\gamma$ reduces only the numerator and therefore has a greater effect. At this relatively low antiviral efficacy ($\varepsilon \approx 0.8$), as the initial number of infectious virions increases and as the establishment probability approaches 100%, differences in the efficacy of antivirals reducing *p*, $\beta$ or $\gamma$ to reduce the establishment probability vanishes (see Supplementary Material, Section S2).

As the antiviral efficacy is increased further, it eventually reaches the critical point where $R_0=1$, and the establishment probability, $(R_0-1)/{\mathcal {B}}$, equals zero. The value of the antiviral efficacy at which this is achieved is the same for antivirals reducing *p* or $\gamma$ (line $\textcircled {1}$ in Fig. 4) since they affect $R_0$ (Eq. (7)) identically. The efficacy of an antiviral reducing $\beta$ must be much higher in order to reach this critical point (line $\textcircled {2}$ in Fig. 4), because reducing $\beta$ reduces both the numerator and denominator of $R_0$.

Therefore, as Czuppon et al.^23^ before us, and for their choice of parameters, we find that at lower efficacies, an antiviral reducing $\beta$ is better than one reducing *p* at reducing the establishment probability, whereas at higher efficacies it is the opposite. However, here we show that an antiviral reducing $\gamma$ at any efficacy is best, better than one reducing *p* or $\beta$, at reducing the establishment probability.

### Distinction between infection extinction and establishment

The mathematical expression for the probability of extinction of an infection, ${\mathcal {P}}_{V \rightarrow \, \text {Extinction}}$, gives the probability, starting from a single infectious virion, that this virion fails to infect a cell, or should it succeed, the probability that this cell’s virion progeny all go on to fail, or should any succeed, the probability that the cells they infect in turn produce virions that all fail, or should any succeed ..., and so on. The recursive nature of this expression makes it difficult to accuratelly define what constitutes the establishment or extinction of an infection in biological terms. Can one look at a given biological or SM infection outcome and classify it as an extinct versus an established infection?

**Figure 5:**
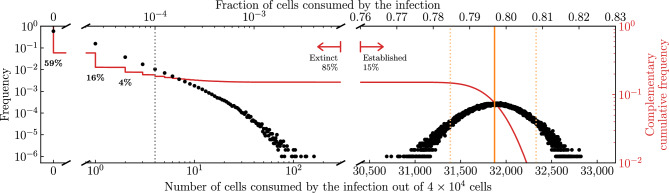
Infection extinction versus establishment. The distribution for the frequency (black dots) or complementary cumulative frequency (solid red curve), or $1-$(cumulative frequency), of the number of cells consumed by the infection, $N_\text {cells}-T(\infty )$, out of $N_\text {cells} = {4 \times 10^4}\,{\text {cells}}$, based on $10^6$ SM simulations. For the frequency distribution, each black dot represents the fraction of the SM simulations where exactly this discrete number of cells were consumed by the infection (e.g. 0, 1, 2, ...). The $95^{\textrm{th}}$-percentile upper and lower bounds (95% bounds) for the number of cells consumed by extinct infections is between zero and the vertical (grey) dotted line, and that by established infections is indicated by a pair of vertical (orange) dashed lines flanking the median (vertical solid line). Parameters are the same as in Fig. 4 for an antiviral reducing the virus production rate, $p\rightarrow (1-\varepsilon )p$, at efficacy $\varepsilon =0.79$ such that $T^{*}/N_\text {cells} = 0.5$ (see Methods).

Let us look at the variety of infection outcomes that the mathematical expression, ${\mathcal {P}}_{V \rightarrow \, \text {Extinction}}$, expects will fall into two categories: established or extinct infections. Figure 5 shows the frequency and complementary cumulative frequency distributions, or $1-$(cumulative frequency), for the number of cells consumed by $10^6$ SM simulated infections. The SM infection outcomes neatly separate into 2 distinct categories: infections that consumed none or very few cells (close to 0%, on the left); and those that consumed many (around 80%, on the right), which we will assume correspond to infections said to have gone extinct or to have established, respectively, as indicated by red arrows. The presumed extinct infections make up 85% (848,227) of the $10^6$ SM simulations, which matches the theoretically predicted probability of infection extinction, ${\mathcal {P}}_{V \rightarrow \, \text {Extinction}}$, for the infection parameters used (see Methods). Of these, 69% (59%/85%) result in no cells consumed, 93% ($(59+16+4)\%/85\%$) in fewer than 3 cells consumed, and 95% in fewer than 5 cells consumed, or less than 0.01% of all susceptible cells. Of the presumed established infections, all consumed at least 30,600 cells or about 10,000$\times$ more than infections considered extinct. The presumed extinct infections are unlikely to engage a measurable immune response nor trigger symptoms, whereas the presumed established infections, consuming  80% of cells, certainly would. In this case, the difference between extinction and establishment is statistically and biologically significant. While 85% of patients would essentially avoid infection altogether, the other 15%, (around 1 in 6) would experience substantial infections.

We show in the Methods (see Eq. (40)) that the median fraction of cells consumed by established infections in the SM monotonically decreases as one increases the quantity $T^{*}/N_\text {cells}$, where

10 $$\begin{aligned} \frac{T^{*}}{N_\text {cells}} = \frac{c/(\beta N_\text {cells}/s)}{\gamma p\tau _I-1} \ . \end{aligned}$$

For the presumed established infections in Fig. 5, Eq. (40) based on $T^{*}/N_\text {cells}$ predicts that $\sim$80% of cells (31,873 out of 40,000) will be consumed, indicated by a vertical solid line in the figure. Biologically, the quantity $T^{*}/N_\text {cells}$ is not particularly meaningful, but mathematically it shows how the median fraction of cells consumed by established infection depends on and is sensitive to each parameter.

**Figure 6:**
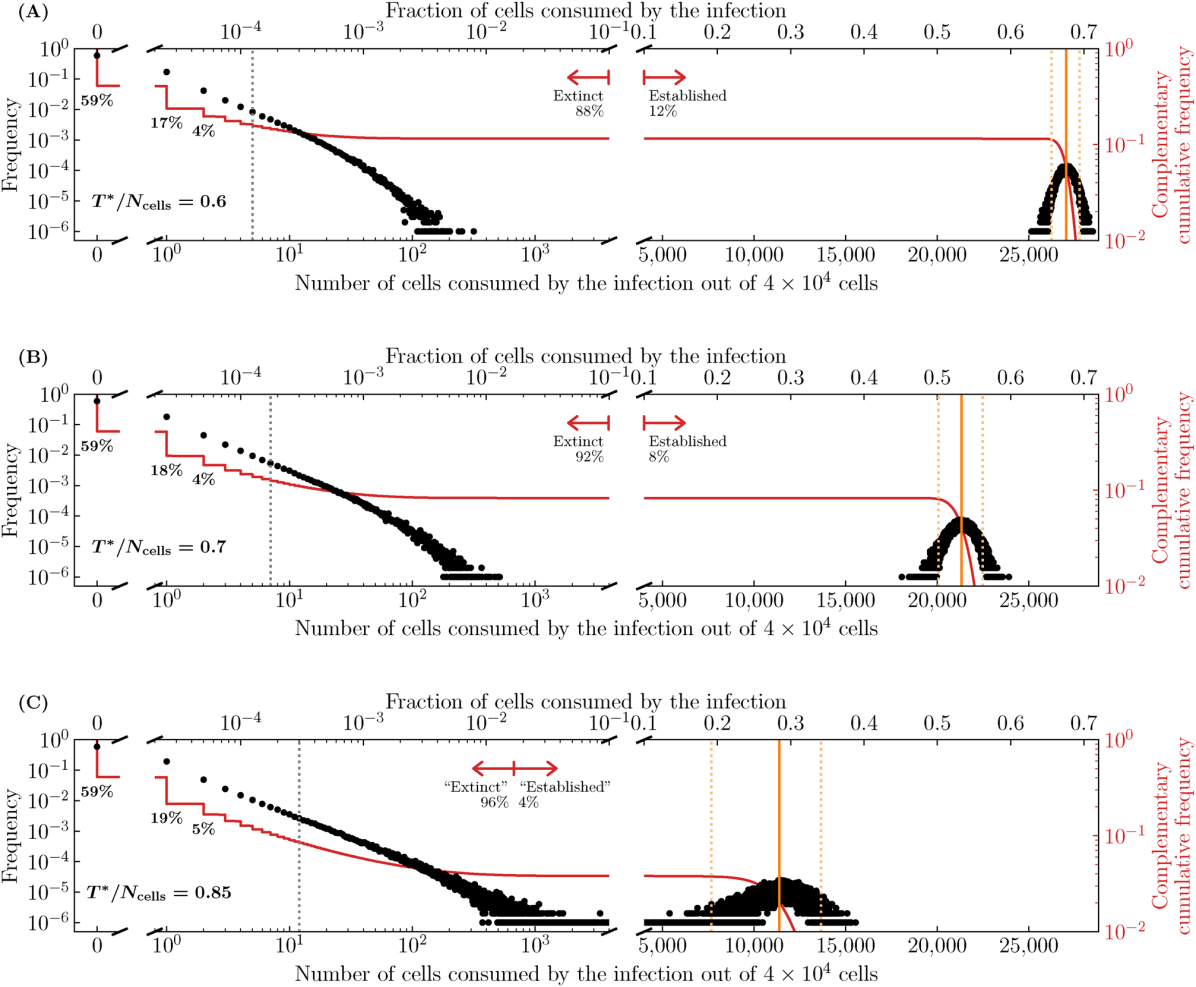
Effect of infection parameters on extinction and establishment. The frequency or complementary cumulative frequency of the fraction or number of cells consumed by the infection out of $N_\text {cells} = {4\times 10^4}\,{\text {cells}}$ where the efficacy ($\varepsilon$) of an antiviral reducing the virus production rate, i.e. $p\rightarrow (1-\varepsilon )p$, was varied such that (**A**) $T^{*}/N_\text {cells} = 0.6$; (**B**) 0.7 or (**C**) 0.85. Everything else is generated, computed, and represented visually as described in the caption of Fig. 5.

Figure 6 shows the frequency and complementary cumulative frequency distributions of the number of cells consumed by an infection, using parameter sets where $T^{*}/N_\text {cells}$ is increasingly closer to 1. It is achieved here by increasing the efficacy of an antiviral reducing the virus production rate, $p\rightarrow (1-\varepsilon )p$, thus increasing $T^{*}/N_\text {cells}$. As $T^{*}/N_\text {cells}$ is increased from 0.6 to 0.85, the median number of cells consumed by infections presumed extinct increases slightly, while that by established infections decreases from $\sim$68% down to $\sim$28%, and the distinction blurs as the two distributions begin to merge. In Fig. 6C, the red arrow demarcating so-called extinct and established infections is located so that $\sim$96% of the $10^6$ SM simulated infections are to the left. Mathematically, this location corresponds to the expected fraction of extinct infections, ${\mathcal {P}}_{V \rightarrow \, \text {Extinction}}$, but the difference in the fraction of cells consumed by established and extinct infections is becoming less biologically meaningful. Therefore, when an antiviral is applied and the establishment probability is non-zero ($R_0>1$), varying the antiviral efficacy affects not only the establishment probability but also the fraction of cells consumed by both established and extinct infections, and likely also the intensity of the immune response and symptoms. For higher antiviral efficacies (higher $T^{*}/N_\text {cells}$), the biological distinction between extinct and established infections becomes increasingly meaningless.

**Figure 7:**
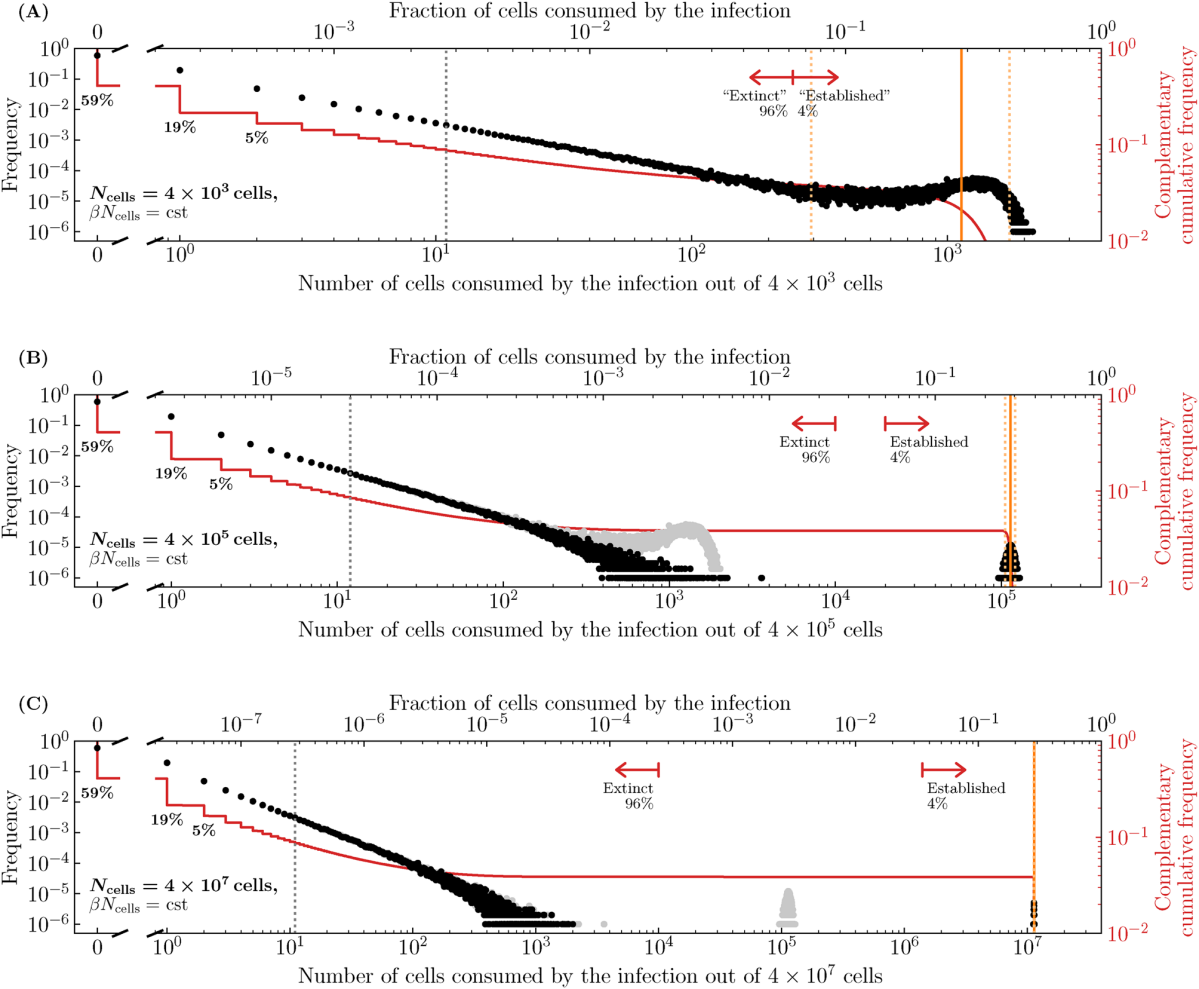
Effect of the susceptible cell population size on extinction and establishment. The frequency and complementary cumulative frequency of the fraction or number of cells consumed by the infection out of $N_\text {cells}$, as the latter is varied, while $\beta N_\text {cells}$, and therefore also the establishment probability and $T^{*}/N_\text {cells}$, are fixed; (**A**) $N_\text {cells} = {4 \times 10^3}\text {cells}$; (**B**) ${4 \times 10^5}\text {cells}$ or (**C**) ${4 \times 10^7}\text {cells}$. The frequency distribution in (**A**) is shown in (**B**) in grey, and that in (**B**) is shown in (**C**) in grey. As in Fig. 6(**C**), the efficacy ($\varepsilon$) of an antiviral reducing the virus production rate, $p\rightarrow (1-\varepsilon )p$, was such that $T^{*}/N_\text {cells} = 0.85$. Everything else is generated, computed, and represented visually as described in the caption of Fig. 5.

The parameter values used in our SM are taken from Czuppon et al.^23^ where the number of cells susceptible to SARS-CoV-2 (parameter $N_\text {cells}$) is assumed to be 40,000 cells, or around just 0.01% of the estimated $4\times 10^8$ cells in the human upper respiratory tract. Yet in earlier work, some of the same authors estimate that a more plausible 1% of these cells (4 million cells) are susceptible to SARS-CoV-2^44^. Mathematically, assuming a larger $N_\text {cells}$ with a proportionally smaller $\beta$ yields the same probability of infection extinction (${\mathcal {P}}_{V \rightarrow \, \text {Extinction}}$) and the same *fraction* but not the same *number* of cells infected by established infections, because it leaves $T^{*}/N_\text {cells}$ but not $T^{*}$ unchanged. Figure 7 explores the fraction and number of cells consumed by infections as the size of the cell population, $N_\text {cells}$, is increased while keeping $\beta N_\text {cells}$ fixed, and thus also the establishment probability and the fraction of cells consumed by established infections. In Fig. 7A, with the smallest cell population considered, the distributions for the number of cells consumed by extinct and established infections are merged, making the two outcomes biologically indistinguishable. In Fig. 7B,C, as the size of the cell population ($N_\text {cells}$) is increased, the fraction of cells consumed by established infections remains centred at $\sim$28% (because $T^{*}/N_\text {cells}$ is unchanged), while the number of cells consumed (28% of $N_\text {cells}$) increases as $N_\text {cells}$ increases, from 4,000 cells up to 40 million cells. Underestimating the number of host cells susceptible to infection, $N_\text {cells}$, can thus have profound implications for the expected severity and extent of immune engagement expected from so-called established infections.

**Figure 8:**
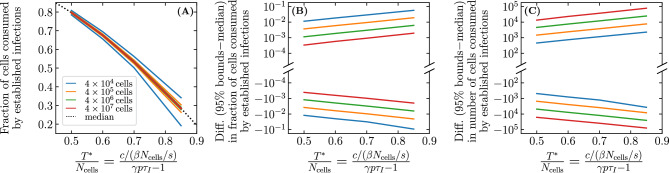
Effect of the susceptible cell population size on cells consumed by infection. The 95% bounds about the median (Eq. (40)) for the fraction and/or number of cells consumed by established infections, as a function of $T^{*}/N_\text {cells}$, for different susceptible cell population sizes ($N_\text {cells}$) while holding $\beta N_\text {cells}$ fixed. The 95% bounds are based on $10^6$ SM simulations of which no less than about 40,000 were established infections (${\mathcal {P}}_{V \rightarrow \, \text {Establishment}}$=4% at $T^{*}/N_\text {cells}=0.85$). Here, $T^{*}/N_\text {cells}=[c/(\beta N_\text {cells}/s)]/[\gamma p\tau _I-1]$ is increased by increasing the efficacy of an antiviral reducing the virus production rate, $p\rightarrow (1-\varepsilon )p$.

Figure 8 explores more generally how the median and 95% bounds of the distribution for the number and fraction of cells consumed by established infections vary as a function of $T^{*}$ and $N_\text {cells}$. Increasing $T^{*}/N_\text {cells}$ from 0.5 to 0.94 decreases the median fraction of cells consumed from 80% down to 12%. Extrapolating from Fig. 8B (see Methods), this corresponds to about zero to 7,600 cells ($[0,0.19]\times N_\text {cells}$) consumed by established infections for $N_\text {cells}=4\times 10^4$, sufficiently few that all extinct and established infections could possibly be asymptomatic. In contrast, for a susceptible cell population of $N_\text {cells}=4\times 10^6$, established infections would consume $\sim [0.11,0.13]\times N_\text {cells}$, or between $\sim$440,000 to 520,000 cells, which are unlikely to be asymptomatic infections. For larger (possibly more realistic) susceptible cell population sizes, the distinction between the fraction of cells consumed by extinct and established infections is greater, and a higher antiviral efficacy (higher $T^{*}/N_\text {cells}$) is required for the distinction to vanish.

### Reduction in the number of cells consumed by infections under antiviral therapy

**Figure 9:**
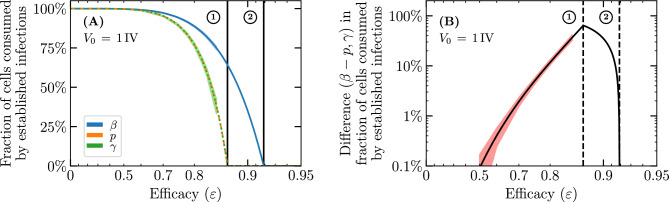
Effect of different antivirals on the fraction of cells consumed by established infections. (**A**) Fraction of cells consumed by established infections initiated with only one infectious virion as a function of efficacy ($\varepsilon$) for antivirals acting prophylactically either to reduce the virus entry rate, $\beta \rightarrow (1-\varepsilon )\beta$, the virus production rate, $p\rightarrow (1-\varepsilon )p$, or the probability of a successful cell infection post virus entry, $\gamma \rightarrow (1-\varepsilon )\gamma$. (**B**) Difference in the fraction of cells consumed by established infections shown in (**A**) for an antiviral reducing $\beta$ minus that for one reducing either *p* or $\gamma$. The vertical lines are the same as in Fig. 4 and indicate antiviral efficacies at which the establishment probability goes to zero ($R_0=1$) for an antiviral reducing *p* or $\gamma$ (line $\textcircled {1}$); or $\beta$ (line $\textcircled {2}$). The thin shaded areas visible over portions of the curves correspond to the 95% bounds about the median over (**A**) 10,000 SM simulations; or (**B**) 10,000 differences between pairs of SM simulations for an antiviral reducing either $\beta$ or $\gamma$, drawn at random with replacement. Infection parameters are the same as in Fig. 4.

As shown above, prophylactic antiviral therapy not only reduces an infection’s establishment probability, but also decreases the fraction of cells consumed by established infections, which decreases as $T^{*}/N_\text {cells}= [c/(\beta N_\text {cells}/s)]/[\gamma p \tau _I -1]$ increases (see Methods, Eq. (40)). Figure 9A shows the fraction of cells consumed by established infection, given an initial inoculum of only one infectious virion, as a function of antiviral efficacy ($\varepsilon$) for antivirals reducing either $\beta$, *p*, or $\gamma$. At a given efficacy, $\varepsilon$, an antiviral reducing *p* or $\gamma$ will increase $T^{*}/N_\text {cells}$ identically because both appear in the same way in $T^{*}/N_\text {cells}$, whereas one reducing $\beta$ will cause a smaller increase in $T^{*}/N_\text {cells}$. As such, an antiviral reducing *p* or $\gamma$ will reduce the fraction of cells consumed by established infections more than one reducing $\beta$. Here again, $\beta$ and *c* appear as a ratio in $T^{*}/N_\text {cells}$, as they did in the infection establishment probability, Eq. (9), such that an antiviral increasing *c* and/or decreasing $\beta$ will have the same impact on reducing the fraction of cells consumed by established infections. Figure 9B illustrates how these differences, and therefore their biological relevance, depends on the antiviral efficacy under consideration. The difference is most pronounced as one approaches the line labelled $\textcircled {1}$ from either side, which corresponds to the efficacy ($\varepsilon \approx 0.87$) at which $R_0=1$ for an antiviral reducing *p* or $\gamma$ while still $R_0>1$ for one reducing $\beta$. The shaded areas on segments of the curves in Fig. 9 correspond to the 95% bounds, and can only be computed from the SM simulations over portions of the curve where established and extinct infections are clearly distinct. It shows that the SM variability is small relative to the difference in the fraction of cells infected.

**Figure 10:**
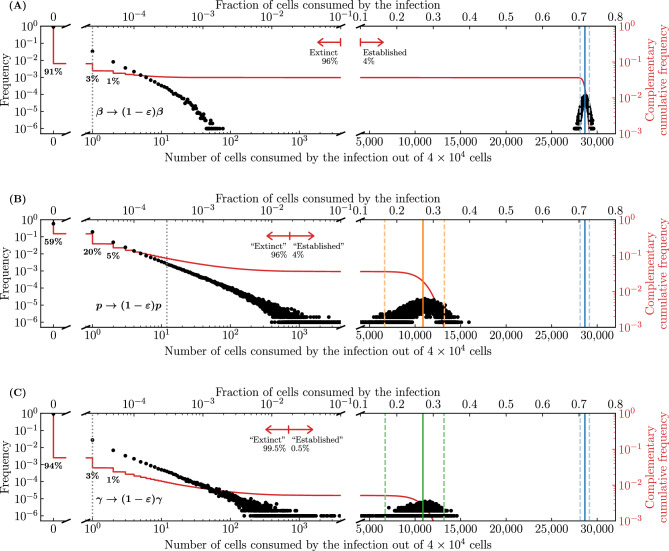
Antiviral with different modes of action cause different fraction of cells to be consumed by infections. The number of cells consumed by the infection out of $N_\text {cells} = {4 \times 10^4}\,\text {cells}$ for antivirals with efficacy $\varepsilon =0.86$ acting either to reduce (**A**) the virus entry rate, $\beta \rightarrow (1-\varepsilon )\beta$, (**B**) the virus production rate, $p\rightarrow (1-\varepsilon )p$, or (**C**) the probability of a successful cell infection post virus entry, $\gamma \rightarrow (1-\varepsilon )\gamma$. Antiviral modes of action are represented by different colours: reducing $\beta$ (blue), *p* (orange) or $\gamma$ (green). Everything else is generated, computed, and represented visually as described in the caption of Fig. 5.

Figure 10 shows the number of cells consumed by extinct and established infections, given an initial inoculum of a single infectious virion, for antivirals reducing $\beta$, *p* or $\gamma$ at an antiviral efficacy of $\varepsilon = 0.86$. This efficacy was chosen so that, given the infection parameters, the establishment probability is the same for an antiviral reducing $\beta$ or *p* (${\mathcal {P}}_{V \rightarrow \, \text {Establishment}}= 4\%$), yet it corresponds to a lower establishment probability for an antiviral reducing $\gamma$ (${\mathcal {P}}_{V \rightarrow \, \text {Establishment}}=0.5\%$). This can be seen from Fig. 4: it corresponds to the efficacy where the orange line (antiviral reducing *p*) crosses the blue line (antiviral reducing $\beta$). At this efficacy, and for the chosen infection parameters, an antiviral reducing $\beta$ compared to one reducing *p* or $\gamma$ will result nearly $\sim$3$\times$ more cells consumed by established infections, and likely engage a more intense immune response and symptoms. There could be clinical merit to weighing a reduction in the probability of infection establishment against a reduction in the severity of those infections that will become established.

We investigated a second parameter set also explored in Czuppon et al.^23^, which is characterized by a 10-fold decrease in the number of susceptible cells $N_\text {cells}$, a 10-fold increase in the virus production rate *p* (and in the average burst size, ${\mathcal {B}} = p \tau _I$), and a corresponding $\sim$10-fold decrease in $\beta N_\text {cells}$ (see Supplementary Material, Section S3). For this second parameter set, we found that an antiviral reducing $\gamma$ was comparable to one reducing $\beta$, but better than one reducing *p*, for reducing the establishment probability, but all 3 modes of action reduced the fraction of cells consumed by established infections by the same amount at the same drug efficacy, $\varepsilon$. Since the degree to which an antiviral reducing $\gamma$ is better than one reducing *p* or $\beta$ depends on infection parameters, it is critical that such parameters be well-determined if such investigations are to yield useful predictions. More generally, we can show that, for infections initiated with one or a few infectious virions, an antiviral *reducing* $\gamma$ *will always be better or at least as good* than one reducing *p* or $\beta$ at reducing the establishment probability and the fraction of cells consumed by established infections *irrespective of the parameters* (see Methods).

Lastly, as in Czuppon et al.^23^, we also considered antiviral therapy for an infection initiated with only one infectious cell which could be representative of post-exposure antiviral therapy (see Supplementary Material, Section S4). In this case, we found that an antiviral reducing *p* or $\gamma$ has the same effectiveness, greater than one reducing $\beta$, in reducing the establishment probability, which differs from the results reported above for an infection initiated with a single infectious virion. In contrast, we found that an antiviral reducing *p* or $\gamma$ reduced the fraction of cells consumed by established infections more so than one reducing $\beta$ at the same efficacy, which exactly matched the results for an infection initiated with only one infectious virion.

Overall, these results over different infection parameters and antiviral efficacy indicates that, under certain conditions, the fraction of cells consumed by both or either extinct and established infections can be an important consideration when evaluating and comparing antivirals with different modes of action. It also highlights the importance of properly identifying biologically relevant base parameter values in order to provide meaningful comparisons.

### Continuous versus burst release of virus and the impact of the virus burst size distribution

**Figure 11:**
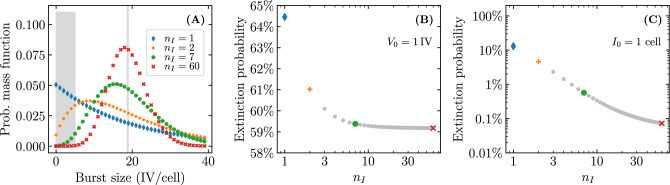
Effect of the infectious phase duration’s shape parameter, $n_I$, on the extinction probability. (**A**) Negative binomial distributed virus burst size as a function of the shape parameter ($n_I$) of the Erlang distributed infectious cell lifespan. The shaded region highlight a region where the burst size is small ($\le 5$ infectious virions) and therefore more likely to result in infection extinction. The vertical grey line corresponds to the average burst size value (${\mathcal {B}} = p \tau _I = {18.8}\,{\text {IV}/\text {cell}}$), which is constant as $n_I$ is varied. (**B**,**C**) The probability of infection extinction as a function of $n_I$, given an infection initiated with only one infectious virion (**B**); or one infectious cell (**C**). Note that the extinction probability is shown on a linear scale in (**B**) but a logarithmic scale in (**C**) to better capture the relationships. Unless otherwise specified, the parameters are the same as in Fig. 4.

In the MFM and SM used herein, virus is released continuously at a fixed rate of *p* infectious virion per hour by infectious cells. In the SM, this fixed rate maps to a Poisson distributed, discrete, stochastic number of infectious virions produced per time step by each infected cell over the duration of its infectious lifespan. The duration of this infectious phase is represented by an Erlang distribution characterized by shape parameter $n_I$ and average duration $\tau _I$. As shown in Eq. (6), this means that the SM’s stochastic virus burst size follows a negative binomial or Pascal distribution with a mean corresponding to the MFM’s virus burst size, ${\mathcal {B}} = p\tau _I$, where $n_I$ now corresponds to the distribution’s integer-valued, stopping-time parameter. Figure 11A illustrates how the SM’s burst size distribution varies as a function of $n_I$, for a fixed average burst size.

Up to this point, as with most SMs, the duration of the infectious phase has been assumed to be exponentially distributed ($n_I = 1$ in Eq. (1)) and, hence, the burst size to be geometrically distributed (special case of the negative binomial distribution). With higher values of $n_I$, the burst size is represented by the more general case of the negative binomial distribution, and as $n_I \rightarrow \infty$, it approaches a Poisson distribution. The choice of $n_I$ does not affect the fraction of cells consumed by established infections, since $n_I$ does not appear in Eq. (40), but it does affect the extinction probability, as per Eq. (8). Previously, Yan et al.^5^ have shown that increasing $n_I$ leads to a decrease in the extinction probability when infection is initiated with a small initial number of infectious virions. Figure 11B shows this for the infection parameters used so far for an infection initiated with a single infectious virion.

Recall that the extinction probability is an implicit expression with 2 main terms,

$$\begin{aligned} {\mathcal {P}}_{V \rightarrow \, \text {Extinction}}= (1-{\mathcal {P}}_{V \rightarrow \, I }) + {\mathcal {P}}_{V \rightarrow \, I }\cdot \sum _{m=0}^\infty \underbrace{\text {NB}\left( m\left| r_\text {fail}=n_I,p_\text {success}=\frac{{\mathcal {B}}}{n_I+{\mathcal {B}}}\right. \right) }_{\text {burst size distribution}} \cdot \left( {\mathcal {P}}_{V \rightarrow \, \text {Extinction}}\right) ^m \ . \end{aligned}$$

where the first term, $1-{\mathcal {P}}_{V \rightarrow \, I }$, is the probability that the initial virion inoculum fails to productively infect a single cell, and the second is the likelihood that it does actually cause a cell infection which then itself fails to establish an infection. Figure 11C shows how the contribution from the second term, i.e. the extinction probability for an infection initiated with a single infected cell, decreases as $n_I$ increases. What is observed in Fig. 11B, therefore, is the shrinking contribution to ${\mathcal {P}}_{V \rightarrow \, \text {Extinction}}$ by the second term as $n_I$ increases.

This second term depends critically on the burst size distribution which, in our SM, is a result of the Erlang distributed infectious phase duration. As the latter distribution goes from exponential ($n_I=1$) to fat-tailed ($n_I\in [2,7]$), to normal-like ($n_I=60$), tending ultimately to a Dirac delta distribution ($n_I\rightarrow \infty$), it becomes less probable that an infectious cell will have a very small burst size, as shown in Fig. 11A. For example, the probability that an infectious cell will have a burst size less than or equal to 5 infectious virions (shaded region in Fig. 11A) is 27% when $n_I=1$ compared to 0.07% when $n_I=60$ ($>380\times$ less likely). This is why, as $n_I$ increases, the probability of infection extinction once one cell has been infected decreases, and this effect is more pronounced for larger virus burst sizes.

**Figure 12:**
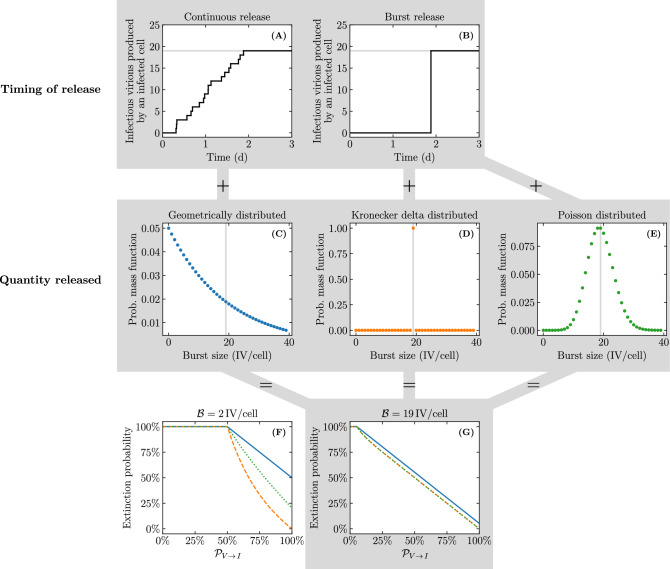
Visual representation for the different modes of virus production explored in Pearson et al.^2^. Two attributes are simultaneously varied for the SMs explored in Pearson et al.^2^: the timing of infectious virion released by infected cells and the total quantity of infectious virions released. Cumulative number of infectious virions released by an infectious cell (**A**) at a constant rate over its lifespan; or (**B**) as a single burst upon its death. Distributions explored for the total number of infectious virions released (burst size, ${\mathcal {B}}$) by a population of infected cells: (**C**) geometric; (**D**) Kronecker delta; or (**E**) Poisson. Pearson et al.^2^ defined continuous production to mean continuous release + geometrically distributed ${\mathcal {B}}$ (A+C); burst production to mean burst release + delta distributed ${\mathcal {B}}$ (B+D); and explored an alternative random burst model with burst release + Poisson distributed ${\mathcal {B}}$ (B+E). The vertical grey line represents the average burst size value ${\mathcal {B}} = {19}\,{\text {IV}/\text {cell}}$. The infection extinction probability for the 3 burst size distributions (**C**,**D**,**E**), as a function of the probability that a single infectious virion productively infects at least one cell (${\mathcal {P}}_{V \rightarrow \, I }$), given an average burst size of (**F**) ${2}\,{\text {IV}/\text {cell}}$; or (**G**) ${19}\,{\text {IV}/\text {cell}}$.

Work by Pearson et al.^2^, expanded in Conway et al.^3^, concluded that the continuous release of infectious virions throughout an infected cell’s lifespan leads to a higher probability of infection extinction than when virions are released as a single burst at the end of a cell’s lifespan^45–48^. In the authors’ continuous production model, infected cells release virions at a constant rate over the duration of their infectious lifespan (Fig. 12A), while in their burst model, virions are released all at once, as a single burst, upon an infected cell’s death (Fig. 12B). The total number of virions thus produced (burst size) is geometrically distributed in their continuous production model (Fig. 12C), and Kronecker delta distributed (a fixed value) in their burst model (Fig. 12D). As such, two attributes were simultaneously varied and conflated: the timing of virus production by infected cells (continuously versus as a burst); and the total number of virions released (burst size). In the end, the difference in extinction probabilities was the result of the different burst size distributions (geometric vs. delta distributed), rather than the timing of the release (continuous vs. burst). As can be seen from Eq. (8), the timing of release does not affect the extinction probability since each infection event, i.e. whether or not each infectious virion results in an infection, can be treated independently of other infectious virions.

Biologically, the mode or timing of the release does not have a one-to-one relationship with the total number of virions that will be released (burst size). In fact, Pearson et al.^2^ considered a variation of their burst model wherein the burst size, released all at once upon the cell’s death, followed a Poisson distribution (Fig. 12E), rather than a Kronecker delta distribution. Figure 12F–G illustrates the differences in the extinction probability for the geometric, Kronecker delta, and Poisson distributed burst sizes explored in Pearson et al.^2^. Echoing earlier results (Fig. 11B), distributions with a higher probability of low burst sizes (geometric followed by Poisson then delta) result in higher extinction probabilities. The effect is most pronounced when the average burst size, ${\mathcal {B}}$, is small.

Having explored how the shape parameter of the infectious phase duration distribution ($n_I$) affects the extinction probability, we can now consider its impact in evaluating and comparing antivirals. For a higher value of $n_I$, since the extinction probability is lower, the establishment probability, or $1-$(extinction probability), is higher. This means that although the effectiveness of antivirals in reducing the establishment probability does not change qualitatively (the better ones remain better), it does change quantitatively (see Supplementary Material Fig. S6). For example, the choice of $n_I$ affects $\varepsilon _{50}$, the efficacy at which the establishment probability is 50% of its value without antivirals, i.e. at $\varepsilon = 0$ (see Table 2). Czuppon et al.^23^ state that for an infection initiated with 10 infectious virions, $\varepsilon _{50}$ is 81% for an antiviral reducing $\beta$, and 85% for an antiviral reducing *p*, where the authors have assumed $n_I=1$. When $n_I = 60$, however, we find that $\varepsilon _{50}$ is comparable for an antiviral reducing $\beta$ vs. *p* (85% vs. 86%), and far less (77%), and therefore potentially easier to achieve, for one reducing $\gamma$. This again highlights the importance of properly estimating parameters before making quantitative comparisons of antiviral regimens.

**Table 2:** Efficacy at which the establishment probability is 50% of its value without antivirals ($\varepsilon _{50}$).

Mode of action	Actual $\varepsilon _{50}$ (relative to $\gamma$)	
	for $n_I =1$	for $n_I = 60$
for an initial inoculum of $V_0=1\,\mathrm{IV}$		
Reducing $\gamma$	43% (0%)	49% (0%)
Reducing $\beta$	57% (14%)	62% (13%)
Reducing *p*	77% (34%)	82% (33%)
for an initial inoculum of $V_0=10\,\mathrm{IV}$		
Reducing $\gamma$	71% (0%)	77% (0%)
Reducing $\beta$	81% (10%)	85% (8%)
Reducing *p*	85% (14%)	86% (9%)

## Discussion

Stochastic models (SMs) of virus infection kinetics usually do not represent failure of a virion to cause an infection post cell entry. Yet biologically, there are many ways in which a virion, post cell entry, will fail to cause a cell infection that will yield infectious progeny. Herein, we constructed a SM of virus infection kinetics with an explicit parameter ($\gamma$) to represent the probability that a virion will cause a productive infection post cell entry. The SM was first used to estimate the extinction probability of an infection, i.e. the probability that an infection will fail to take hold or spread significantly.

Previously, Czuppon et al.^23^ compared prophylactic antivirals reducing virus entry ($\beta$) or production (*p*) for their ability to reduce the establishment probability, or $1-$(extinction probability), of a SARS-CoV-2 infection in patients. Extending this work, we investigated how an antiviral reducing $\gamma$ would compare against antivirals reducing $\beta$ or *p*. We found that a prophylactic antiviral reducing $\gamma$ was best at reducing the establishment probability when infection is initiated with a small number of infectious virions. When instead an infection is initiated with an initial number of infectious cells, possibly representative of post-exposure antiviral therapy, we found that an antiviral reducing $\gamma$ or *p* caused the same reduction in the establishment probability, better than that for an antiviral reducing $\beta$. While the degree to which an antiviral with a particular mode of action is better than another depends on the chosen parameters, under prophylactic therapy our SM predicts that an antiviral reducing $\gamma$ will invariably reduce the probability of establishment *at least as much or more so*, than one reducing $\beta$ or *p* with the same efficacy, irrespective of the choice of parameters.

In HIV antiviral therapy, reverse transcriptase inhibitors (RTIs) prevent the transcription of viral DNA from viral RNA, a step that occurs after virus entry and is necessary for replication. Conway et al.^3^ have reported that, under pre-exposure antiviral therapy, RTIs which they represented as reducing their SM’s cell infection rate ($\beta$) are more effective than protease inhibitors (reducing the virus production rate, *p*), at reducing the risk of HIV infection. By adding $\gamma$ in their expression for the extinction probability, we can show that an antiviral reducing $\gamma$, which better captures the mode of action of a RTI, rather than $\beta$ is even more effective at reducing the risk of infection for pre-exposure antiviral therapy, for the infection parameter sets explored. Irrespective of the parameters considered, *reducing* $\gamma$ *will always be as effective or more so* in this respect. These findings (detailed in Supplementary Material, Section S5) echo those reported herein for SARS-CoV-2 parameters, as to the equal or better performance of antivirals reducing $\gamma$, compared to *p* or $\beta$. The introduction of parameter $\gamma$ to capture productive cell infection failure *after* an infectious virion has successfully entered a cell is an important consideration when comparing antivirals based on their mode of action.

When the SM was used to simulate infections under various conditions and therapeutic efficacies, the infection outcomes at low antiviral efficacies separated into two clear groups: infection of none or very few cells; and infection of many or most cells. The proportion of infections resulting in nearly no cells consumed, presumed to be the result of infection extinction, indeed matched the infection extinction probability derived for our SM. For infections where many or most cells were consumed, presumed to be established infections, we derived a theoretical expression for the median fraction of cells consumed, and its dependence on the SM parameters. Antiviral therapy acting on a particular infection parameter reduced both the infection’s establishment probability and the number of cells consumed by presumed established infections. This finding had 2 important implications. Firstly, since the infection’s establishment probability and the number of cells consumed by established infections depend differently on the SM’s parameters, different antiviral modes of action decreased one quantity more effectively than the other. Secondly, and perhaps more importantly, as antiviral efficacy increases, the fraction of cells consumed by established infections decreases, eventually reaching a point where the fraction consumed by established and extinct infections becomes indistinguishable. For example, at a drug efficacy that yields an equal probability of infection extinction, we found that an antiviral reducing $\beta$, compared to one reducing *p*, resulted in $\sim$3$\times$ more cells consumed by so-called established infections. At that same efficacy, an antiviral reducing $\gamma$ yielded both a lower probability of infection establishment, and resulted in fewer cells consumed overall. Since even an infection extinction probability of 99% will lead to infection establishment in one person out of a hundred, reducing the severity of this infection should be an important clinical consideration when comparing antivirals.

Infection severity often correlates, or is assumed to correlate, with peak or total viral load^49–52^, this is not always the case^53,54^, and different measures such as the degree of lung involvement can be better indicators^55,56^. For example, Myers et al.^55^ stained whole-lung sections of IAV infected mice for influenza nucleoprotein to quantify the % active (antigen-positive) and inactive (lesioned and minimally antigen-positive) area. This quantity is analogous to the fraction of cells consumed by infections tracked herein. Myers et al.^55^ showed that the measured % total lesioned (active + inactive) lung area nonlinearly correlates with the % weight loss, an indicator of disease severity. Looking at the probability that infections will consume more than some biologically critical number of cells, e.g. that identified as sufficient to trigger an immune response and its associated symptoms or minimally sufficient for transmission, could provide a more appropriate target against which to select optimal antiviral candidates.

To facilitate direct comparison of our work herein to past work by others, most of our results were based on assuming that the lifespan of infectious cells, the period during which an infected cell is releasing virus, is exponentially distributed (Erlang distributed with shape parameter $n_I=1$). But the lifespan of infectious cells for many different viruses has been shown in vitro to be inconsistent with an exponential distribution, and instead follow a more normal-like distribution ($n_I>\sim$10). Yan et al.^5^ have shown previously that increasing $n_I$ leads to a decrease in the extinction probability. Herein we established that this happens because the probability that an infectious cell will randomly produce a very small total number of virions (burst size) becomes vanishingly small as $n_I$ increases. We showed that while $n_I$ does not affect the fraction of cells consumed by established infections, increasing $n_I$ decreases the probability of infection extinction by decreasing the likelihood of very small burst sizes, thus requiring higher antiviral efficacy to achieve the same risk reduction.

Previous work by Pearson et al.^2^, expanded in Conway et al.^3^, states that continuous virus production and release over a infected cell’s lifespan leads to a higher risk of infection extinction than when virus is released all at once as a burst upon an infected cell’s death^45–48^. Herein we demonstrated that it is the burst size distribution (total number of virions released by infected cells), and not the mode of release (continuous vs all at once), that determines the probability of infection extinction. We explored why the mode or timing of the release (continuous vs burst) does not imply a specific burst size distribution.

The SM introduced herein is general and, like the mean-field mathematical model on which it is based, it could be applicable to a wide range of different viruses. It does, however, make a number of simplifying assumptions that might limit the scope of its applicability. We list some of those below, and discuss how they might impact the findings reported herein.

*Immune response and cell regeneration* Our results, along with nearly all previous work mentioned herein which used SMs to study virus infection establishment versus extinction focused on in vivo rather than in vitro infections. More accurately, the parameters of these SMs were chosen so as to recapitulate in vivo infection kinetics, but did not include cell regeneration nor an explicit immune response (except for Yan et al.^5^). For established infections, which consume a much higher number of cells, the immune response likely plays an important role in clearing the infection, and towards the end of the infection cell regeneration could begin to replenish the target cell population. Both could likely translate into fewer cells consumed by established infection such that the distinction in the fraction of cells consumed by extinct versus established infections could vanish at even lower antiviral efficacies.

*Well-mixed assumption versus spatial inhomogeneity* Our SM assumes that the virus and susceptible cells constitute a well-mixed system: all cells are exposed to the same virus concentration. The assumption is reasonable for infection in a liquid medium, for viruses whose primary mode of dissemination is through cell-free dissemination to susceptible cells^27^. But a number of viruses disseminate exclusively or primarily via direct, cell-to-cell contact, possibly via or as the cause of syncytia formation^57–60^. Compared to cell-free infection, cell-to-cell infection can be more rapid, more likely to be successful due to transfer of multiple virus genomes, and can offer protection against extracellular defenses (e.g. neutralizing antibodies)^61^. The well-mixed assumption might also hold for a mixed population of cells with differing susceptibility for the virus, where our SM would represent the average susceptibility across all cell types. But the specific spatial arrangement of susceptible cells, or large inhomogeneity in the concentration of virus over the space covered by susceptible cells, could result in significant deviations of the infection kinetics from the idealized, well-mixed assumption. For example, in certain solid organs, tissues, or in vitro organoids where cells are organized as layers, as the infection progresses, susceptible cells from the upper layers die and detach, revealing fresh susceptible cells from lower layers, or causing differentiation of other cells into susceptible cells. Such complex interactions and geometries would require tailored modification of the SM presented herein, and detailed, often unavailable knowledge of the parameters required to describe these more complicated arrangements. For IAV infection within the human respiratory tract, we previously used a mathematical model to show how the rapid mucus escalator-induced advection of virions released from the lower respiratory tract upwards towards the nose and mouth, has a strong protective effect. By sweeping virus away from cells in lower sites towards those in upper sites, advection partly helped infection to establish more easily and proceed more rapidly in upper sites, while providing a high degree of protection to lower sites^62^. It would be interesting to re-visit this past work in a stochastic context to compare it against the results presented herein.

*Defective interfering and semi-infectious particles* Our SM explicitly represents virion that successfully enter a cell but fail to cause an infection, but assumes this process leaves the cell in the same state as if entry had not occurred. Biologically, repeated virus failure post cell entry would likely trigger antiviral pathways within the cell, leaving it in a different state than its naive, susceptible state. Additionally, the accumulation of failed virions within a cell over a sufficiently short time could eventually add up to one functional productive infection (e.g., semi-infectious particles with an incomplete set of segmented genomes rescuing one another^40,41^) or lead to defective interfering particle production^42^.

*Virion pleomorphism* Our SM does not consider the impact of virus pleomorphism^63–66^. For RSV, the pattern observed in vitro for the rate of loss of virion infectivity could be explained by the combined slow versus rapid loss of infectivity by two distinct virus populations, possibly filamentous versus spherical particles^67^. For IAV and IAV pseudotyped with Ebola virus surface proteins, filamentous particles were observed in vitro to be less sensitive than spherical particles to neutralizing antibodies or fusion inhibitors^68^. It is possible that representing the diverse population of individual virions as more or less identical virions whose average properties represent the average over all the subpopulations it contains provides a suitable approximation of this diversity. If two very distinct subpopulations exist, it is also possible that the most infectious population largely determines the infection kinetics which would then also be well represented based on a single homogeneous virus population.

Generally, it is unclear how important these various factors are to shaping the course of an infection, and thus the extent to which they need to be represented. Very simple models in the past have proven to be very effective at not only reproducing, but also predicting the course and outcome of infections both in vitro and in vivo^33,62,69,70^. This suggests that under physiologically relevant conditions, at least some of these factors can be safely neglected or implicitly represented. With regards to the specific results presented herein, the probability that the infection will become extinct depends on the first few infection events, and if the infection does become extinct very few cells will get consumed. Hence, it is reasonable to expect that the immune response and cell regeneration would not significantly affect the extinction probability or the fraction of cells consumed by extinct infections. Similarly, with so few virions produced by extinct infections, cell co-infection or re-infection is improbable, such that semi-infectious and defective interfering particles would have a negligible impact, the dominant virus subpopulation would determine the outcome, only cells from upper layers of a tissue or organ would see the virus, and spatial inhomogeneity might not yet have developed. For established infections, undoubtedly at least some of these processes would significantly impact the overall fraction of cells consumed. If, however, they did so equally over all antiviral modes of action, these processes might only alter the precise fraction of cells consumed by each mode, and not the more general findings herein as to which mode of action constitutes the most suitable target.

## Methods

### Parameter values used to generate all figures

The following are the parameters used to generate each figure found within the manuscript.

**Figure 13:**
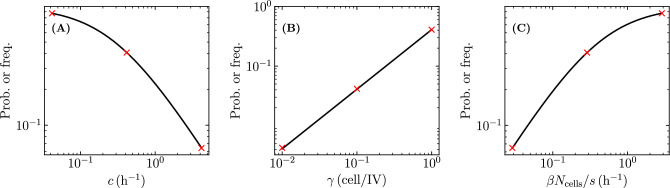
Probability that an infectious virion is successful at causing a productive cell infection. Frequency of success of an infectious virion to cause a productive cell infection in a population of uninfected cells for $10^5$ SM simulations (red scatter) compared to ${\mathcal {P}}_{V \rightarrow \, I }$ the derived expression for the probability of success of an infectious virion to cause a productive cell infection in a population of uninfected cells (black curve, Eq. (19)) while either varying (**A**) *c*, (**B**) $\gamma$ or (**C**) $\beta N_\text {cells}/s$. Unless otherwise specified, the parameters were the same as in Fig. 4.

**Figure 14:**
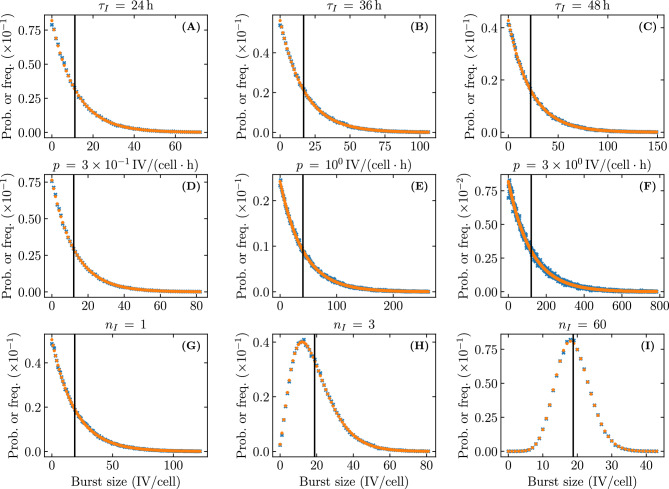
Probability that an infectious cell will produce *m* infectious virions. Normalized histogram of the burst size, i.e. the total number of infectious virions produced by one infectious cell over its lifespan, for $10^5$ SM simulations (blue x), compared to the derived expression for the probability mass function of the burst size distribution (orange circle, Eq. (21)) while either varying (**A**–**C**) $\tau _I$, (**D**–**F**) *p* or (**G**–**I**) $n_I$. The black lines show the average burst size values ${\mathcal {B}} = p \tau _I$. Unless otherwise specified, the parameters were the same as in Fig. 4.

**Figure 15:**
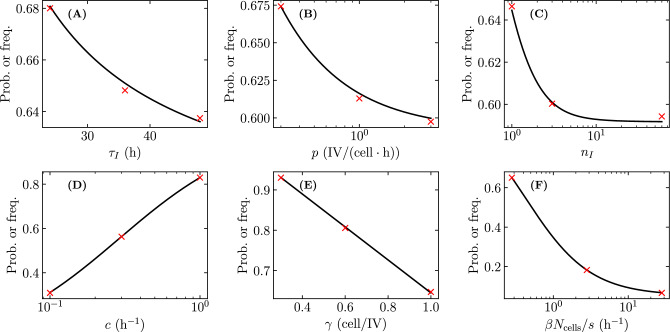
Extinction probability given an infection initiated with only one infectious virion. Frequency of failure of an infection initiated with only one infectious virion to cause more than 0.01% of cells to be infected by the end for $10^5$ SM simulations (red scatter) compared to ${\mathcal {P}}_{V \rightarrow \, \text {Extinction}}$ the derived expression for the extinction probability given an infection initiated with only one infectious virion (black curve, Eq. (30)) while either varying (**A**) $\tau _I$, (**B**) *p*, (**C**) $n_I$, (**D**) *c*, (**E**) $\gamma$ or (**F**) $\beta N_\text {cells}/s$. Unless otherwise specified, the parameters were the same as in Fig. 4.

- Figures 4, 13, 14, 15$p = {(11.2/24)}\,{\text {IV}/(\text {cell} \cdot \,\text {h})}$, $N_\text {cells} = {4\times 10^4}\,{\text {cells}}$, $R_0 = 7.69$, $\tau _E = {24/5}\,{\text {h}}$, $\tau _I = {(24/0.595)}\,{\text {h}}$, $c = {(10/24)}\,{\text {h}^{-1}}$, $n_E = 1$, $n_I = 1$ and $\beta = [cR_0/\tau _I]/[N_\text {cells}(p-R_0/\tau _I)]$^23^. Also, $\gamma = {1}\,{\text {cell}/\text {IV}}$ and $s= {1}{\text {mL}}$.
- Figure 5 As in Fig. 4 but with $p \rightarrow (1-\varepsilon )p$, where $\varepsilon =0.79$.
- Figure 6 As in Fig. 5 but with $\varepsilon = 0.82,\,0.84,\,0.86$.
- Figure 7 As in Fig. 5 but with $\varepsilon = 0.86$, $N_\text {cells} = {4 \times [10^3,10^5,10^7]}\,{\text {cells}}$ and $\beta N_\text {cells}$ fixed.
- Figure 8 As in Fig. 5 but with $\varepsilon = 0.79,\,0.82,\,0.84,\,0.86$, $N_\text {cells} = {4 \times [10^4,10^5,10^6,10^7]}\,{\text {cells}}$ and $\beta N_\text {cells}$ fixed. Extrapolation of 95% bounds discussed in the text was done by performing linear regression of the $\log _{10}$ of the difference (95% bounds—median) in fraction of cells consumed presented in Fig 8B.
- Figure 9 As in Fig. 4 but SM simulations with $\varepsilon = 0.50,\,0.60,\,0.70,\,0.75,\,0.78,\,0.80,\,0.82,\,0.83,\,0.85$.
- Figure 10 As in Fig. 4 but with an antiviral acting either to reduce $\beta$, *p* or $\gamma$, where $\varepsilon = 0.86$.
- Figure 11 As in Fig. 4 but with $n_I = 1,\,2,\,7,\,60$.
- Figure 12$${\mathcal {B}} = {19}\,{\text {IV}/\text {cell}}$$.
- Figure 17A As in Fig. 4 but with $\beta \rightarrow (1-\varepsilon )\beta$, where $\varepsilon = 0.81$.

**Figure 16:**
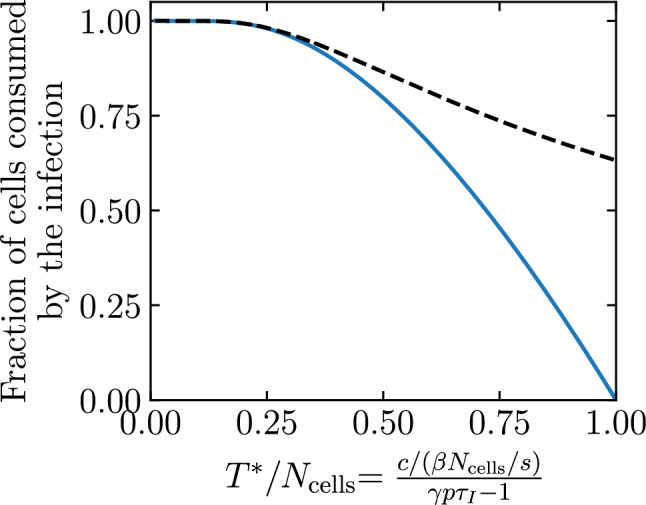
Fraction of cells consumed by infections in the MFM. The fraction of cells consumed by infection in the MFM ($1-T(\infty )/N_\text {cells}$, Eq. (42), blue solid line) as a function of the fraction of cells uninfected when the reproductive number $R(t)=1$ ($T^{*}/N_\text {cells}$). The black dashed line is $1-\exp [-1/(T^{*}/N_\text {cells})]$.

**Figure 17:**
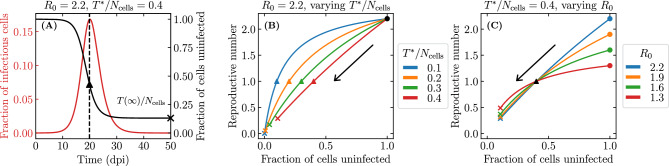
Exploring the critical fraction of uninfected cells, $T^{*}/N_\text {cells}$. (A) MFM time courses for the fraction of uninfected (black) and infectious (red) cells, for an infection initiated with 10 infectious virions under antiviral therapy reducing the virus entry rate, $\beta \rightarrow (1-\varepsilon )\beta$, at efficacy $\varepsilon = 0.81$, which yields an establishment probability (${\mathcal {P}}_{V \rightarrow \, \text {Establishment}}=48\%$) that is $\sim$50% of its value without antivirals^23^. The point $(t,T^{*}/N_\text {cells})$ is represented by a triangle; the time when $T = T^{*}$ by a dashed line; and $T=T(\infty )$ by an $\times$ on the right vertical axis. The parameters are provided in Methods, but notably $n_I=1$. (B,C) The reproductive number, *R*(*t*), as a function of the fraction of cells that remain uninfected, *T*(*t*), over the course of an infection (time is implicit), based on Eq. (48) when either $R_0 = 2.2$ and $T^{*}/N_\text {cells}$ is varied (B); or $T^{*}/N_\text {cells} = 0.4$ and $R_0$ is varied (C). The start of the infection is represented by a circle; the critical point where $T=T^{*}$ and $R = 1$ by a triangle; and the end of the infection where $T=T(\infty )$ as given by Eq. (42) by an $\times$.

Figures 16 and 17B,C are generated using Eqs. (42) and (48), neither of which depend directly on infection parameters, but instead depend on $T^{*}/N_\text {cells}$ and/or $R_0$ whose value is specified thereon.

### Details of the SM implementation

The SM is given by Eq. (1), where the changes in the SM variables are random whole numbers drawn at each time step, as follows:

- $E_i^\text {out} = \text {Binomial}(n=E_i^t,\, p_E= \Delta t\cdot n_E/\tau _E)$ corresponds to the number of cells in the $i^\text {th}$ eclipse compartment ($E_i$) that will transition to the $(i+1)^\text {th}$ compartment over a time interval $\Delta t$, given that there are $E_i^t$ cells in the $i^\text {th}$ compartment at time step *t*. Each of the $E_i^t$ cells in eclipse compartment *i* undergo an independent Bernoulli trial with two possible outcomes: either the cell transitions to the $(i+1)^\text {th}$ compartment with success probability $p_E=\Delta t\cdot n_E/\tau _E$, or otherwise remains in the $i^\text {th}$ compartment. The time step, $\Delta t$ is chosen to be sufficiently small to ensure $p_E < 1$, as discussed below.
- $I_j^\text {out} = \text {Binomial}(n=I_j^t,\, p_I= \Delta t\cdot n_I/\tau _I)$ is the number of cells in the $j^\text {th}$ infectious compartment ($I_j$) that transition to the $(j+1)^\text {th}$ compartment over a time interval $\Delta t$, given that there are $I_j^t$ cells in the $j^\text {th}$ compartment at time step *t*, and the probability of transition $p_I= \Delta t\cdot n_I/\tau _I$.
- $V^\text {prod} = \text {Poisson}(\lambda =\Delta t\cdot p\,\sum _{j=1}^{n_I} I_j^t)$ is the number of infectious virions newly produced into the supernatant over a time $\Delta t$, given that there are $\sum _{j=1}^{n_I} I_j^t$ infectious cells at time step *t*, each producing infectious virions at a rate of *p* infectious virions per hour. The Poisson distributed random variable, $V^\text {prod}$ is the number of events that occurred, given the expected number of occurrences over a time $\Delta t$, namely $\lambda =\Delta t\cdot p\sum _{j=1}^{n_I} I_j^t$.
- $V^\text {decay},V^\text {enter},\text {otherwise} = \text {Trinomial}(n=V^t,\, p_1=\Delta t\cdot c,\, p_2=\Delta t\cdot \beta T^t/s,\, p_3=1-p_1-p_2)$ corresponds to the number of infectious virions in the supernatant at time *t*, $V^t$, that end up in each of 3 possible fates. $V^\text {decay}$ is the number of infectious virions that lose infectivity with probability $p_1=\Delta t\cdot c$, $V^\text {enter}$ are lost from the supernatant as they enter a target cell with probability $p_2=\Delta t\cdot \beta T^t/s$, and otherwise infectious virions do neither and remain in the supernatant with probability $p_3=1-p_1-p_2$. Each probability ($p_1,p_2,p_3$) can be considered constant over time interval $\Delta t$, provided a sufficiently small time step is chosen, as discussed below.
- $N^\text {inf} = |\{x_i | x_i \in {\mathcal {U}}\{a=1,\, b=T^t\}, 1 \le i \le V^\text {suc}\}|$ is the number of target cells that become infected ($T\rightarrow E_1$) over time $\Delta t$, given that $V^\text {enter}$ infectious virions enter into target cells, out of which $V^\text {suc} = \text {Binomial}(n=V^\text {enter},\, p_V=\gamma )$ infectious virions ultimately lead to successful cell infection, given probability $p_V=\gamma$. Random variable $N^\text {inf}$ does not correspond to any named probability mass function. The $N^\text {inf}$ expression simulates randomly placing $V^\text {suc}$ infectious virions into $T^t$ cells chosen at random with replacement, $\{x_i | x_i \in {\mathcal {U}}\{a=1,\, b=T^t\}, 1 \le i \le V^\text {suc}\}$, and counts the number of distinct cells that received one or more infectious virions, $|\{\text {...}\}|$. Computationally, it is implemented in python as len(numpy.unique(numpy.random.choice(a=${T}^\texttt {t}$,size=${V}^\text {suc}$,replace=True))).

The duration of the SM’s discrete time steps, $\Delta t$, which sets the probability of event occurrences or the number of such events, is computed at each iteration step *t* as

11 $$\begin{aligned} \Delta t = \frac{{\mathcal {P}}_{\text {events}}}{ \max \bigg \{ \frac{\beta T^t}{s}, c, \frac{n_E}{\tau _E}, \frac{n_I}{\tau _I} \bigg \} } \end{aligned}$$

where ${\mathcal {P}}_{\text {events}}$ is the probability of occurrence of the most likely event, namely of virion loss due to cell entry ($\beta T^t/s$) or loss of infectivity (*c*), or transition of cells from one infected state to another ($n_E/\tau _E$ and $n_I/\tau _I$). A value of ${\mathcal {P}}_{\text {events}}=0.05$ (or 5%) is used, as it was found to be sufficiently small to provide accurate solutions (see “Numerical accuracy of the SM’s solutions” in Methods).

Some readers might prefer to see the SM expressed as transitions^2,5^, namely12One limitation of this representation is that it indicates that one virion that enters a cell and that is successful at causing an infection with rate $\gamma \beta /s$ will cause the infection of one cell﻿ ($V\,+\,T\,\rightharpoonup\,E_1$). In reality, in our SM, one successful virion does not necessarily cause the infection of one cell (see $N^\text {inf}$ in Table 1) because one cell could receive two successful infectious virions which would only result in one rather than two new successful cell infections.

Our stochastic modelling approach is similar to standard applications of the $\tau$-leaping method^71^. One important difference is that the $\tau$-leaping method represents the number of events that will occur for each reaction as a Poisson random variable. Because there is no upper bound on a Poisson distributed random variable, this assumption can lead to negative populations of reactants when the number of events that cause the loss of a reactant are larger than the number of reactants available^72–74^. To address this issue, variations of the $\tau$-leaping method make use of finite range binomial^72,73^ or multinomial^74^ random variables, as was done herein.

Our SM expression for the number of target cells that become infected, $N^\text {inf}$, as a function of the number of virion entries that successfully lead to cell infection ($V^\text {suc}$) is novel. It differs from previous work that assumed as many infected cells as successful virions entries ($N^\text {inf}=V^\text {suc}$) by instead correctly accounting for cases when more than one successful virions enter the same cell leading to a single cell infection ($N^\text {inf}\le V^\text {suc}$). In contrast, our theoretical expressions for the extinction probability, Eq. (2), and the median fraction of cells consumed by established infections, Eq. (40), assume $N^\text {inf}=V^\text {suc}$, as in previous work. Yet in cases where infection extinction and establishment are clearly distinct, the proportion of SM infections presumed extinct agrees with the theoretically predicted extinction probability, and the median fraction of cells consumed by SM infections presumed established agrees well with our theoretical expression. This is likely because the number of infectious virions $V^\text {suc}$ considered herein is sufficiently small compared to the number of cells $T^t$ that the entry of more than one $V^\text {suc}$ into the same $T^t$ cell occurs rarely and $N^\text {inf}=V^\text {suc}$ largely holds for the parameters explored herein. Nevertheless, our SM’s expression for $N^\text {inf}$ more accurately represents the underlying process, and its computational cost is negligible, and thus worth keeping.

The MFM was developed independently from the SM. As such, the MFM was not intended to be exactly the same as the mean of the SM equations. If one wanted to derive the mean of the SM equations, one would have to consider higher order moments of the SM variables^75–77^. Using a simplified version of our SM, we derived second order moment equations, approximated third order moments, and found no significant disagreement between the MFM solution with and without second order moment terms (see Supplementary Material, Section S1).

### Numerical accuracy of the SM’s solutions

SM simulation output presented in Results, for both extinct and established infections, are those associated with Figs. 5, 6, 7, 10, i.e. those showing the frequency distribution for the number of cells consumed by $10^6$ SM simulated infections. The most important quantities discussed about these figures are the simulated fraction of extinct infections and median fraction of cells consumed by established infections. These can be compared to the theoretical extinction probability, ${\mathcal {P}}_{V \rightarrow \, \text {Extinction}}$, and median fraction of cells consumed by established infections, $1-T(\infty )/N_\text {cells}$. When extinction and establishment are clearly distinct, the absolute error of ${\mathcal {P}}_{V \rightarrow \, \text {Extinction}}$ and $1-T(\infty )/N_\text {cells}$ is of the order of $10^{-4}$ or 0.01% (see Table 3 and 4). Further, the absolute error of ${\mathcal {P}}_{V \rightarrow \, \text {Extinction}}$ is always within one standard deviation of the expected distribution for the fraction of extinct infections after $10^6$ SM simulations, $1/10^6 \cdot \text {Binomial}(n=10^6,\, p = {\mathcal {P}}_{V \rightarrow \, \text {Extinction}})$ (see Table 3). This indicates that the time step criteria (Eq. (11)) and the number of SM simulations were sufficient to provide good accuracy for our key results.

**Table 3:** Absolute errors for the extinction probability.

Figures	${\mathcal {P}}_{V \rightarrow \, \text {Extinction}}^\text {Theoretical}$	${\mathcal {P}}_{V \rightarrow \, \text {Extinction}}^\text {Simulated}$	Standard deviation$^\dagger$	Absolute error
5	0.848170	0.848227	0.000359	0.000057
6(A)	0.884467	0.884348	0.000320	0.000119
6(B)	0.917389	0.917172	0.000275	0.000213
7(B)	0.961389	0.961308	0.000193	0.000081
7(C)	0.961389	0.961223	0.000193	0.000166
10(A)	0.963306	0.963198	0.000188	0.000108

$$^\dagger$$ Standard deviation = $$1/10^6 \cdot \sqrt{10^6\cdot {\mathcal {P}}_{V \rightarrow \, \text {Extinction}}^\text {Theoretical} \cdot \left( 1-{\mathcal {P}}_{V \rightarrow \, \text {Extinction}}^\text {Theoretical}\right) }$$

**Table 4:** Absolute errors for the median fraction of cells consumed by established infections.

Figures	$\left[ 1-T(\infty )/N_\text {cells}\right] ^\text {Theoretical}_\text {Established, median}$	$\left[ 1-T(\infty )/N_\text {cells}\right] ^\text {Simulated}_\text {Established, median}$	Absolute error
5	0.796818	0.796850	0.000032
6(A)	0.675769	0.675850	0.000081
6(B)	0.533028	0.533325	0.000297
7(B)	0.284271	0.284442	0.000171
7(C)	0.284271	0.284187	0.000084
10(A)	0.716323	0.716375	0.000052

The numerical solution of the SM was further validated by replacing the SM’s random variables, e.g. $E_i^\text {out}$, by their expected value, e.g. $E_i^\text {out} = E_i^t\cdot \Delta t\cdot n_E/\tau _E$, and comparing the solution against that obtained for the MFM in Eq. (1) with a standard numerical ODE solver. The solutions were found to be in agreement over the wide range of parameter values explored (not shown).

### MFM using the expected value of the SM’s random variables

One way of defining a MFM is by replacing the random variables of the SM by their expected values, listed in Table 5. This is trivial for the random variables that are drawn from well-defined probability distributions. But it is not for $N^\text {inf}$, which is determined by simulating the process of randomly placing $V^\text {suc}$ infectious virions into $T^t$ cells chosen at random with replacement, $\{x_i | x_i \in {\mathcal {U}}\{a=1,\, b=T^t\}, 1 \le i \le V^\text {suc}\}$, and counting the number of distinct cells that received one or more infectious virions, $|\{\text {...}\}|$.

Consider then $e_n$, the expected number of empty cells after placing *n* infectious virions into $T^t$ cells. After placing $n-1$ infectious virions into $T^t$ cells, there is on average $e_{n-1}$ empty cells by definition. There is then a $e_{n-1}/T^t$ probability to place the last infectious virion into an empty cell. It follows that

13 $$\begin{aligned} e_n&= \frac{e_{n-1}}{T^t}(e_{n-1}-1) + \bigg (1-\frac{e_{n-1}}{T^t}\bigg ) e_{n-1} \nonumber \\ e_n&= \frac{e^2_{n-1}}{T^t}-\frac{e_{n-1}}{T^t}+e_{n-1}-\frac{e^2_{n-1}}{T^t} \nonumber \\ e_n&= e_{n-1} \bigg (\frac{T^t-1}{T^t} \bigg ) \end{aligned}$$

By recursion and since trivially $e_0\,=\,T^t$,

14 $$\begin{aligned} e_n&= T^t \bigg (\frac{T^t-1}{T^t} \bigg )^n \end{aligned}$$

The expected number of $N^\text {inf}$ cells after placing $V^\text {suc}$ infectious virions into $T^t$ cells is therefore represented as

15 $$\begin{aligned} T^t \Bigg [1-\bigg (\frac{T^t-1}{T^t} \bigg )^{V^\text {suc}} \Bigg ] \end{aligned}$$

When there are far fewer successful virion entry events than there are target cells ($V^\text {suc} \ll T^t$), the above expression for the expected value of $N^\text {inf}$ equals $V^\text {suc}$.

**Table 5:** Expected value of the random variables used by the SM.

Random variable	Expected value
$E_i^\text {out}$	$E_i^t \cdot \Delta tn_E/\tau _E$ where $i=1,2,...,n_E$
$I_j^\text {out}$	$I_j^t \cdot \Delta tn_I/\tau _I$ where $j=1,2,...,n_I$
$V^\text {prod}$	$\Delta t\cdot p\,\sum _{j=1}^{n_I} I_j^t$
$V^\text {decay},V^\text {enter},\text {otherwise}$	$V^t \cdot \Delta tc,V^t \cdot \Delta t\beta T^t/s, V^t \cdot (1-\Delta tc-\Delta t\beta T^t/s)$
$N^\text {inf}$	$T^t\big [1-[(T^t-1)/T^t]^{V^\text {suc}} \big ]$ for $T^t \ge 1$ and 0 for $T^t < 1$
	where the expected value of $V^\text {suc}$ is $V^{\text {enter}} \cdot \gamma$

### Probability that an infectious virion succeeds at causing a productive cell infection

Let us derive the probability that an infectious virion is successful at causing a productive cell infection, ${\mathcal {P}}_{V \rightarrow \, I }$, in a population of fully susceptible, uninfected cells ($T=N_\text {cells}$).

In a time step $\Delta t$, the probability that an infectious virion neither loses infectivity nor enters a cell is $(1-\Delta tc-\Delta t \beta N_\text {cells}/s)$, the probability that an infectious virion enters a cell is $\Delta t\beta N_\text {cells}/s$ and the probability that an infectious virion post cell entry will be successful at causing a cell infection is $\gamma$. The probability that an infectious virion is successful at causing a productive cell infection in time $t\,=\,k\Delta t$ is then given by the following expression,

16 $$\begin{aligned} (1-\Delta tc-\Delta t\beta N_\text {cells}/s)^{k-1}(\Delta t \beta N_\text {cells}/s)\gamma \end{aligned}$$

More generally, the probability that this happens in any time $t = k\Delta t$ where $0 \le t \le t_\text {incub}$ and $t_\text {incub}\,=\,n\Delta t$ is the incubation time, is then expressed as17In our SM, $\Delta t$ is set according to Eq. (11). Let us assume that $\Delta t$ is sufficiently small that we can take the limit of Eq. (17) as $n \rightarrow \infty$ (i.e. $\Delta t \rightarrow 0$), namely,

18 $$\begin{aligned}&\left[ \frac{\gamma \beta N_\text {cells}/s}{c+\beta N_\text {cells}/s}\right] \left[ 1-\lim _{n \rightarrow \infty }\left( 1-\frac{t_\text {incub}(c+\beta N_\text {cells}/s)}{n}\right) ^n\right] \nonumber \\&\quad = \left[ \frac{\gamma \beta N_\text {cells}/s}{c+\beta N_\text {cells}/s}\right] \left[ 1-\textrm{e}^{-t_\text {incub}(c+\beta N_\text {cells}/s)}\right] \end{aligned}$$

As $t_\text {incub} \rightarrow \infty$, Eq. (18) is then given by the following expression,

19 $$\begin{aligned} {\mathcal {P}}_{V \rightarrow \, I }= \frac{\gamma \beta N_\text {cells}/s}{c+\beta N_\text {cells}/s} = \frac{\gamma }{1+c/(\beta N_\text {cells}/s)} \end{aligned}$$

Fig. 13 shows that the frequency of success of an infectious virion to cause a productive cell infection in a population of uninfected cells, generated from $10^5$ SM simulations, is in agreement with Eq. (19), over a wide range of infection parameters. This means that our earlier oassumption was reasonable that $\Delta t$ is sufficiently small that we can take the limit as $\Delta t \rightarrow 0$.

### Probability that an infectious cell produces *m* infectious virions

Let us derive the probability that an infectious cell produces *m* infectious virions over its lifespan (${\mathcal {P}}_{I \rightarrow \, m \, V}$).

Assuming that successive infectious virion productions are independent events that happen under constant rate *p*, then the probability that an infectious cell produces *m* infectious virions over its lifespan *t* is given by $\text {Poisson}(m|\lambda = t\,p)$.

The negative binomial (NB) distribution represents the probability that there are *k* successes in a sequence of independent and identically distributed Bernoulli trials with probability of success *p* before a specified number of failures *r* occurs, namely,

20 $$\begin{aligned} \text {NB}(k|r,\,p) = {k+r-1 \atopwithdelims ()k}(1-p)^rp^k \end{aligned}$$

The probability that an infectious cell transition to the next compartment is given by $\text {Binomial}(1|n=1,\, p_I= \Delta tn_I/\tau _I)$. Therefore the probability that an infectious cell transition to the next compartment for $n_I$ time steps and does not for $n-n_I$ time steps is then given by $\text {NB}(n_I|r=n-n_I,\, p_I=\Delta t\, n_I/\tau _I)$. It is possible to show that the continuous analogue of this discrete probability distribution is the Erlang distribution ($\text {Erlang}(t|k=n_I,\, \lambda = n_I/\tau _I)$). As a result, we make use of this probability density function to express the probability that the infectious lifespan has duration *t*.

The probability that an infectious cell produces *m* infectious virions over its lifespan, i.e. that the burst size of an infected cell is *m*, can then be derived by integrating over time *t*, the joint probability distribution that an infectious cell produces *m* infectious virions given a lifespan of duration *t* and that an infectious cell has a lifespan of duration *t*, namely,

21 $$\begin{aligned} {\mathcal {P}}_{I \rightarrow \, m \, V}= \int _0^\infty \text {Poisson}(m|\lambda =pt) \cdot \text {Erlang}(t|k=n_I,\, \lambda =n_I/\tau _I) \text {d}t = \text {NB}(m|r=n_I,\, p_B={\mathcal {B}}/(n_I+{\mathcal {B}})) \end{aligned}$$

where ${\mathcal {B}} = p\tau _I$ is the average burst size. When $n_I = 1$, ${\mathcal {P}}_{I \rightarrow \, m \, V}= \text {NB}(m|r=1,\, p_B={\mathcal {B}}/(1+{\mathcal {B}})) = \text {Geom}(m|p=1/(1+{\mathcal {B}}))$ where Geom represents the geometric distribution. When $n_I \rightarrow \infty$, ${\mathcal {P}}_{I \rightarrow \, m \, V}= \text {NB}(m|r=n_I,\, p_B={\mathcal {B}}/(n_I+{\mathcal {B}})) = \text {Poisson}(m|\lambda = {\mathcal {B}})$.

Figure 14 shows that the normalized histogram of the burst size, generated from $10^5$ SM simulations, is in agreement with Eq. (21), over a wide range of infection parameters.

### Extinction probability

Following Pearson et al.^2^, let us derive the extinction probability of an infection. The extinction probability of an infection with initially one infectious virion (${\mathcal {P}}_{V \rightarrow \, \text {Extinction}}$) can be written as

22 $$\begin{aligned} {\mathcal {P}}_{V \rightarrow \, \text {Extinction}}= (1-{\mathcal {P}}_{V \rightarrow \, I })+{\mathcal {P}}_{V \rightarrow \, I }\cdot {\mathcal {P}}_{I \rightarrow \, \text {Extinction}} \end{aligned}$$

since the infection can become extinct if the initial infectious virion fails to cause a productive cell infection with probability $1-{\mathcal {P}}_{V \rightarrow \, I }$ or if it does cause a productive cell infection with probability ${\mathcal {P}}_{V \rightarrow \, I }$ but that cell infection then leads to extinction with probability ${\mathcal {P}}_{I \rightarrow \, \text {Extinction}}$.

Likewise, the extinction probability of an infection with initially one infectious cell (${\mathcal {P}}_{I \rightarrow \, \text {Extinction}}$) can be expressed as

23 $$\begin{aligned} {\mathcal {P}}_{I \rightarrow \, \text {Extinction}}= \sum _{m=0}^\infty {\mathcal {P}}_{I \rightarrow \, m \, V}\cdot \left( {\mathcal {P}}_{V \rightarrow \, \text {Extinction}}\right) ^m \end{aligned}$$

since the initial infectious cell will produce some number *m* of infectious virions over its lifespan with probability ${\mathcal {P}}_{I \rightarrow \, m \, V}$ and each one of these produced infectious virions can be seen as an independent infection event that can lead to extinction with probability ${\mathcal {P}}_{V \rightarrow \, \text {Extinction}}$.

Substituting our derived expression for ${\mathcal {P}}_{I \rightarrow \, m \, V}$ (Eq. 21) into Eq. (23) yields

24 $$\begin{aligned} {\mathcal {P}}_{I \rightarrow \, \text {Extinction}}&= \sum _{m=0}^\infty \text {NB}(m|r=n_I,p_B={\mathcal {B}}/(n_I+{\mathcal {B}})) \cdot \left( {\mathcal {P}}_{V \rightarrow \, \text {Extinction}}\right) ^m \nonumber \\ {\mathcal {P}}_{I \rightarrow \, \text {Extinction}}&= \sum _{m=0}^\infty {m+n_I-1 \atopwithdelims ()m}\left[ 1-\frac{{\mathcal {B}}}{n_I+{\mathcal {B}}}\right] ^{n_I}\left[ \frac{{\mathcal {B}}}{n_I+{\mathcal {B}}}\right] ^m \cdot \left( {\mathcal {P}}_{V \rightarrow \, \text {Extinction}}\right) ^m \nonumber \\ {\mathcal {P}}_{I \rightarrow \, \text {Extinction}}&= \left[ \frac{n_I}{n_I+{\mathcal {B}}}\right] ^{n_I} \sum _{m=0}^\infty {m+n_I-1 \atopwithdelims ()m}\left[ \frac{{\mathcal {B}}}{n_I+{\mathcal {B}}}\cdot {\mathcal {P}}_{V \rightarrow \, \text {Extinction}}\right] ^m \end{aligned}$$

The following binomial series can be used to simplify Eq. (24),

25 $$\begin{aligned} \frac{1}{(1-x)^s} = \sum _k {s+k-1\atopwithdelims ()k}x^k \end{aligned}$$

Let us derive some related expressions,

26 $$\begin{aligned} x&{:=}\frac{{\mathcal {B}}}{n_I+{\mathcal {B}}}\cdot {\mathcal {P}}_{V \rightarrow \, \text {Extinction}}\end{aligned}$$

27 $$\begin{aligned} \Longrightarrow 1-x&= \frac{n_I+{\mathcal {B}} (1-{\mathcal {P}}_{V \rightarrow \, \text {Extinction}})}{n_I+{\mathcal {B}}} \nonumber \\ \Longrightarrow \frac{1}{(1-x)^{n_I}}&= \left[ \frac{n_I+{\mathcal {B}}}{n_I+{\mathcal {B}} (1-{\mathcal {P}}_{V \rightarrow \, \text {Extinction}})} \right] ^{n_I} \end{aligned}$$

Eq. (24) can then be simplified to

28 $$\begin{aligned} {\mathcal {P}}_{I \rightarrow \, \text {Extinction}}&= \left[ \frac{n_I}{n_I+{\mathcal {B}}}\right] ^{n_I} \left[ \frac{n_I+{\mathcal {B}}}{n_I+{\mathcal {B}} (1-{\mathcal {P}}_{V \rightarrow \, \text {Extinction}})}\right] ^{n_I} \nonumber \\&= \left[ \frac{n_I}{n_I+{\mathcal {B}} (1-{\mathcal {P}}_{V \rightarrow \, \text {Extinction}})}\right] ^{n_I} \nonumber \\&= \left[ \frac{{\mathcal {B}} (1-{\mathcal {P}}_{V \rightarrow \, \text {Extinction}})}{n_I}+1\right] ^{-n_I} \end{aligned}$$

Substituting Eq. (28), our derived expression for ${\mathcal {P}}_{V \rightarrow \, I }$ (Eq. (19)) into Eq. (22) yields

29 $$\begin{aligned} {\mathcal {P}}_{V \rightarrow \, \text {Extinction}}&= (1-{\mathcal {P}}_{V \rightarrow \, I })+{\mathcal {P}}_{V \rightarrow \, I }\left[ \frac{{\mathcal {B}} (1-{\mathcal {P}}_{V \rightarrow \, \text {Extinction}})}{n_I}+1\right] ^{-n_I} \end{aligned}$$

30 $$\begin{aligned} {\mathcal {P}}_{V \rightarrow \, \text {Extinction}}&= \left[ 1-\frac{\gamma \beta N_\text {cells}/s}{c+\beta N_\text {cells}/s}\right] +\frac{\gamma \beta N_\text {cells}/s}{c+\beta N_\text {cells}/s} \left[ \frac{{\mathcal {B}} (1-{\mathcal {P}}_{V \rightarrow \, \text {Extinction}})}{n_I}+1\right] ^{-n_I} \nonumber \\ {\mathcal {P}}_{V \rightarrow \, \text {Extinction}}&= 1-\frac{\gamma }{c/(\beta N_\text {cells}/s)+1}+\frac{\gamma }{c/(\beta N_\text {cells}/s)+1}\left[ \frac{{\mathcal {B}} (1-{\mathcal {P}}_{V \rightarrow \, \text {Extinction}})}{n_I}+1\right] ^{-n_I} \nonumber \\ 0&= 1-\frac{[1+c/(\beta N_\text {cells}/s)]}{\gamma }[1-{\mathcal {P}}_{V \rightarrow \, \text {Extinction}}]-\left[ \frac{{\mathcal {B}} (1-{\mathcal {P}}_{V \rightarrow \, \text {Extinction}})}{n_I}+1\right] ^{-n_I} \end{aligned}$$

This expression does not seem to have an analytical solution for ${\mathcal {P}}_{V \rightarrow \, \text {Extinction}}$ but a solution can be found numerically by finding the roots of the expression on the right-hand side of Eq. (30) using scipy.optimize.fsolve. Figure 15 shows that the frequency of extinction given an infection initiated with only one infectious virion, generated from $10^5$ SM simulations, is in agreement with the probability of extinction given an infection initiated with only one infectious virion, over a wide range of infection parameters. The extinction probability given any number of infectious virions $V_0$ and infectious cells $I_0$ is given by $\left( {\mathcal {P}}_{V \rightarrow \, \text {Extinction}}\right) ^{V_0}\cdot \left( {\mathcal {P}}_{I \rightarrow \, \text {Extinction}}\right) ^{I_0}$. It is important to note that, as others^2,5^, we also make the assumption in this derivation that $T = N_\text {cells}$ is constant.

When $n_I = 1$, Eq. (29) simplifies to

31 $$\begin{aligned} {\mathcal {P}}_{V \rightarrow \, \text {Extinction}}&= (1-{\mathcal {P}}_{V \rightarrow \, I })+{\mathcal {P}}_{V \rightarrow \, I }\left[ \frac{1}{1+{\mathcal {B}} (1-{\mathcal {P}}_{V \rightarrow \, \text {Extinction}})}\right] \end{aligned}$$

In this case, ${\mathcal {P}}_{V \rightarrow \, \text {Extinction}}= (1-{\mathcal {P}}_{V \rightarrow \, I })+1/{\mathcal {B}}$ is a solution to the above expression as,

$$\begin{aligned} (1-{\mathcal {P}}_{V \rightarrow \, I })+1/{\mathcal {B}}&= (1-{\mathcal {P}}_{V \rightarrow \, I })+{\mathcal {P}}_{V \rightarrow \, I }\left[ \frac{1}{1+{\mathcal {B}} [1-(1-{\mathcal {P}}_{V \rightarrow \, I }+1/{\mathcal {B}})]}\right] \\ (1-{\mathcal {P}}_{V \rightarrow \, I })+1/{\mathcal {B}}&= (1-{\mathcal {P}}_{V \rightarrow \, I })+{\mathcal {P}}_{V \rightarrow \, I }\left[ \frac{1}{{\mathcal {B}} \cdot {\mathcal {P}}_{V \rightarrow \, I }}\right] \\ (1-{\mathcal {P}}_{V \rightarrow \, I })+1/{\mathcal {B}}&= (1-{\mathcal {P}}_{V \rightarrow \, I })+1/{\mathcal {B}} \\ \therefore \text {LHS}&= \text {RHS} \end{aligned}$$

${\mathcal {P}}_{V \rightarrow \, \text {Extinction}}= (1-{\mathcal {P}}_{V \rightarrow \, I })+1/{\mathcal {B}}$ is equivalent to Eq. (15) in Pearson et al.^2^. ${\mathcal {P}}_{V \rightarrow \, \text {Extinction}}= (1-{\mathcal {P}}_{V \rightarrow \, I })+1/{\mathcal {B}}$ can also be written in terms of the basic reproductive number $R_0 = {\mathcal {B}} \cdot {\mathcal {P}}_{V \rightarrow \, I }$ (see Eq. (7)), i.e. ${\mathcal {P}}_{V \rightarrow \, \text {Extinction}}= 1-(R_0-1)/{\mathcal {B}}$. Also, ${\mathcal {P}}_{V \rightarrow \, \text {Establishment}}$ or $1 - {\mathcal {P}}_{V \rightarrow \, \text {Extinction}}$ is then given by ${\mathcal {P}}_{V \rightarrow \, I }-1/{\mathcal {B}}$ or $(R_0-1)/{\mathcal {B}}$.

When $n_I \rightarrow \infty$, ${\mathcal {P}}_{I \rightarrow \, m \, V}= \text {Poisson}(m|\lambda = {\mathcal {B}})$, therefore, we obtain,

32 $$\begin{aligned} {\mathcal {P}}_{V \rightarrow \, \text {Extinction}}= (1-{\mathcal {P}}_{V \rightarrow \, I })+{\mathcal {P}}_{V \rightarrow \, I }\cdot \sum _{m=0}^\infty \text {Poisson}(m|\lambda ={\mathcal {B}}) \cdot \left( {\mathcal {P}}_{V \rightarrow \, \text {Extinction}}\right) ^m \end{aligned}$$

which is equivalent to Eq. (26) in Pearson et al.^2^.

### Expression for the fraction of cells consumed by infections

As it happens, the median fraction of cells consumed by established infections in the SM approximately corresponds to the fraction of cells consumed in the deterministic MFM for a given parameter set. We can use the latter to obtain an analytical expression for the median fraction of cells infected by our SM to understand how each SM parameter affects this quantity.

So let us derive the fraction of cells consumed in the MFM for an infection starting with a number of infectious virions *V*(0). Following^78^, we have the following relations from Eq. (1),

33 $$\begin{aligned} {\dot{T}}+\sum _{i=1}^{n_E} {\dot{E}}_i+\sum _{j=1}^{k}{\dot{I}}_j = -n_II_k/\tau _I&\Longrightarrow I_k = -(\tau _I/n_I)\left[ {\dot{T}}+\sum _{i=1}^{n_E} {\dot{E}}_i+\sum _{j=1}^{k}{\dot{I}}_j\right] \end{aligned}$$

34 $$\begin{aligned} \frac{\text {d}}{\text {d}t} \ln (T) = \frac{{\dot{T}}}{T} = -\gamma \beta V/s&\Longrightarrow \frac{1}{\gamma \beta /s}\frac{\text {d}}{\text {d}t}\ln (T) = -V \end{aligned}$$

Substituting these relations into Eq. (1) for ${\dot{V}}$, we obtain

35 $$\begin{aligned} {\dot{V}}&= -(p\tau _I/n_I)\left[ n_I\left( {\dot{T}}+\sum _{i=1}^{n_E}{\dot{E}}_i\right) +\sum _{k=1}^{n_I}\sum _{j=1}^k {\dot{I}}_j\right] +\frac{c}{\gamma \beta /s}\frac{\text {d}}{\text {d}t} \ln (T)+{\dot{T}}/\gamma \end{aligned}$$

Integrating Eq. (35) from 0 to $+\infty$ and using the fact that $E_i(0) = E_i(\infty ) = I_j(0) = I_j(\infty ) = V(\infty ) = 0$ for $i=1,2,...,n_E$ and $j=1,2,...,n_I$, it follows that,

36 $$\begin{aligned} -V(0) = -p\tau _I \left\{ T(\infty )-T(0)\right\} +\frac{c}{\gamma \beta /s}\left\{ \ln [T(\infty )]-\ln [T(0)]\right\} +(1/\gamma )\left\{ T(\infty )-T(0)\right\} \end{aligned}$$

Dividing by $T(0) = N_\text {cells}$ we have

37 $$\begin{aligned} -V(0)/N_\text {cells}&= (1/\gamma -p\tau _I)[T(\infty )/N_\text {cells}-1]+\frac{c}{\gamma \beta N_\text {cells}/s}\ln (T(\infty )/N_\text {cells}) \nonumber \\ -V(0)/N_\text {cells}+1/\gamma -p\tau _I&= (1/\gamma -p\tau _I)T(\infty )/N_\text {cells}+\frac{c}{\gamma \beta N_\text {cells}/s}\ln (T(\infty )/N_\text {cells}) \nonumber \\ -\gamma V(0)/N_\text {cells}+1-\gamma p\tau _I&= (1-\gamma p\tau _I)T(\infty )/N_\text {cells}+\frac{c}{\beta N_\text {cells}/s}\ln (T(\infty )/N_\text {cells}) \nonumber \\ -\frac{\gamma \beta /s\cdot V(0)}{c} -\frac{(\beta N_\text {cells}/s)\cdot (\gamma p\tau _I-1)}{c}&= -\frac{(\beta N_\text {cells}/s)\cdot (\gamma p\tau _I-1)}{c}T(\infty )/N_\text {cells}+\ln (T(\infty )/N_\text {cells}) \end{aligned}$$

Introducing a new quantity we call the critical fraction of cells uninfected, $T^{*}/N_\text {cells} = [c/(\beta N_\text {cells}/s)] / [\gamma p\tau _I-1]$, into Eq. (37) yields

38 $$\begin{aligned} -\frac{\gamma \beta /s\cdot V(0)}{c} -\frac{1}{T^{*}/N_\text {cells}}&= -\frac{T(\infty )/N_\text {cells}}{T^{*}/N_\text {cells}}+\ln (T(\infty )/N_\text {cells}) \nonumber \\ \textrm{e}^{-[\gamma \beta /s\cdot V(0)]/c} \textrm{e}^{-1/(T^{*}/N_\text {cells})}&= T(\infty )/N_\text {cells} \cdot \textrm{e}^{-(T(\infty )/N_\text {cells})/(T^{*}/N_\text {cells})} \nonumber \\ -\frac{\textrm{e}^{-1/(T^{*}/N_\text {cells})}}{T^{*}/N_\text {cells}}\textrm{e}^{-[\gamma \beta /s\cdot V(0)]/c}&= -\frac{T(\infty )/N_\text {cells}}{T^{*}/N_\text {cells}} \cdot \textrm{e}^{-(T(\infty )/N_\text {cells})/(T^{*}/N_\text {cells})} \end{aligned}$$

Eq. (38) is of the form $z = w\exp (w)$. The Lambert *W* function is the function that gives the inverse relation, i.e. $W(z) = w$. Therefore, we have the following,

39 $$\begin{aligned} -\frac{T(\infty )/N_\text {cells}}{T^{*}/N_\text {cells}} = W_0\hspace{-0.3em}\left( -\frac{\textrm{e}^{-1/(T^{*}/N_\text {cells})}}{T^{*}/N_\text {cells}}\textrm{e}^{-[\gamma \beta /s\cdot V(0)]/c}\right) \nonumber \\ T(\infty )/N_\text {cells} = -T^{*}/N_\text {cells} \cdot W_0\hspace{-0.3em}\left( -\frac{\textrm{e}^{-1/(T^{*}/N_\text {cells})}}{T^{*}/N_\text {cells}}\textrm{e}^{-[\gamma \beta /s\cdot V(0)]/c}\right) \end{aligned}$$

where here we make use of $W_0$ the upper branch of the Lambert *W* function. The upper branch is used because $T(\infty )/T^{*}< 1$ since $T(\infty )$ is always reached after $T^{*}$ and therefore is always smaller.

The fraction of cells consumed by the infection, $1-T(\infty )/N_\text {cells}$, is then given by

40 $$\begin{aligned} 1-T(\infty )/N_\text {cells} = 1+T^{*}/N_\text {cells} \cdot W_0\hspace{-0.3em}\left( -\frac{\textrm{e}^{-1/(T^{*}/N_\text {cells})}}{T^{*}/N_\text {cells}}\textrm{e}^{-[\gamma \beta /s\cdot V(0)]/c}\right) \end{aligned}$$

If instead there was initially a number of infectious cells but no infectious virions ($I_1(0) \ne 0$, $V(0) = 0$) then Eq. (36) would be

41 $$\begin{aligned} 0 = -p\tau _I \left\{ T(\infty )-T(0)-I_1(0)\right\} +\frac{c}{\gamma \beta /s}\left\{ \ln [T(\infty )]-\ln [T(0)]\right\} +(1/\gamma )\left\{ T(\infty )-T(0)\right\} \end{aligned}$$

which is equivalent to Eq. (36) where *V*(0) is replaced with $p \tau _I I_1(0)$. This means that Eq. (40) would be the same but with *V*(0) replaced with $p \tau _I I_1(0)$.

For *V*(0) such that $[\gamma \beta /s\cdot V(0)]/c \approx 0$, Eq. (40) simplifies to

42 $$\begin{aligned} 1-T(\infty )/N_\text {cells} = 1+T^{*}/N_\text {cells} \cdot W_0\hspace{-0.3em}\left( -\frac{\textrm{e}^{-1/(T^{*}/N_\text {cells})}}{T^{*}/N_\text {cells}}\right) \end{aligned}$$

The Maclaurin series of $W_0(x)$ is $W_0(x) = x-x^2+3x^3/2-\dots \approx x$. It follows that, when $T^{*}/N_\text {cells} \ll 1$, Eq. (42) simplifies to

43 $$\begin{aligned}&1-T(\infty )/N_\text {cells} \approx 1+T^{*}/N_\text {cells} \cdot -\frac{\textrm{e}^{-1/(T^{*}/N_\text {cells})}}{T^{*}/N_\text {cells}} \nonumber \\&1-T(\infty )/N_\text {cells} \approx 1-\textrm{e}^{-1/(T^{*}/N_\text {cells})} \end{aligned}$$

Figure 16 shows that the fraction of cells consumed by the infection, $1-T(\infty )/N_\text {cells}$, is simply a strictly decreasing function of $T^{*}/N_\text {cells}$ and that, when $T^{*}/N_\text {cells} \ll 1$, $1-T(\infty )/N_\text {cells}\approx 1-\textrm{e}^{-1/(T^{*}/N_\text {cells})}$. But what does $T^{*}/N_\text {cells}$ represent? At the start of an infection, all cells are uninfected, $T(0) = N_\text {cells}$, and the reproductive number, *R*(0), i.e. the number of successful secondary infections caused by an infected cell over its lifespan, corresponds to the basic reproductive number ($R_0$), given by Eq. (7), where $R_0>1$ for an infection that has a non-zero establishment probability. As the infection progresses and fewer uninfected cells remain, each infectious virion has fewer opportunities to cause a successful cell infection and *R*(*t*) decreases.

Let us derive an expression for the reproductive number *R*(*t*) as a function of the fraction of cells uninfected $T(t)/N_\text {cells}$ for a given basic reproductive number $R_0$ (Eq. (7)). To begin, using Eq. (7) and the left-hand side of Eq. (10), we have the following relation,

44 $$\begin{aligned} \frac{1}{R_0}\frac{p\tau _I \cdot \gamma \beta N_\text {cells}/s}{c+\beta N_\text {cells}/s}&=\frac{p\tau _I \cdot \gamma \beta T^{*}/s}{c+\beta T^{*}/s} \nonumber \\ \frac{1}{R_0}\frac{N_\text {cells}}{c/(\beta /s)+N_\text {cells}}&=\frac{T^{*}}{c/(\beta /s)+T^{*}} \nonumber \\ N_\text {cells}[c/(\beta /s)]+N_\text {cells}T^{*}&= R_0[c/(\beta /s)]T^{*}+R_0N_\text {cells}T^{*}\nonumber \\ c/(\beta /s)\cdot (N_\text {cells}-R_0T^{*})&= N_\text {cells}T^{*}(R_0-1) \nonumber \\ c/(\beta /s)&= \frac{N_\text {cells}T^{*}(R_0-1)}{N_\text {cells}-R_0T^{*}} \end{aligned}$$

The left-hand side of Eq. (10) can be rearranged to

45 $$\begin{aligned} \gamma p\tau _I&= \frac{c+\beta T^{*}/s}{\beta T^{*}/s} \nonumber \\ \gamma p\tau _I&= \frac{c/(\beta /s)+T^{*}}{T^{*}} \end{aligned}$$

Substituting Eq. (44) into Eq. (45), we obtain

46 $$\begin{aligned} \gamma p\tau _I&= \frac{1}{T^{*}}\left[ \frac{N_\text {cells}T^{*}(R_0-1)}{N_\text {cells}-R_0T^{*}}+T^{*}\right] \nonumber \\ \gamma p\tau _I&= \frac{N_\text {cells}(R_0-1)}{N_\text {cells}-R_0T^{*}}+1 \nonumber \\ \gamma p\tau _I&= \frac{R_0(N_\text {cells}-T^{*})}{N_\text {cells}-R_0T^{*}} \end{aligned}$$

The reproductive number can also be written as

47 $$\begin{aligned} R(t)&= \frac{p\tau _I \cdot \gamma \beta T(t)/s}{c+\beta T(t)/s} \nonumber \\ R(t)&= \frac{\gamma p\tau _I \cdot T(t)}{c/(\beta /s)+T(t)} \end{aligned}$$

Substituting Eqs. (44) and (45) into Eq. (47), we then have

48 $$\begin{aligned} R(t)&= \frac{\frac{R_0(N_\text {cells}-T^{*})}{N_\text {cells}-R_0T^{*}} \cdot T(t)}{\frac{N_\text {cells}T^{*}(R_0-1)}{N_\text {cells}-R_0T^{*}}+T(t)} \nonumber \\ R(t)&= \frac{R_0 T(t)\cdot (N_\text {cells}-T^{*})}{N_\text {cells}T^{*}(R_0-1)+T(t) \cdot (N_\text {cells}-R_0T^{*})} \nonumber \\ R(t)&= \frac{R_0 \cdot T(t)/N_\text {cells} \cdot (1-T^{*}/N_\text {cells})}{T^{*}/N_\text {cells} \cdot (R_0-1)+T(t)/N_\text {cells} \cdot (1-R_0 \cdot T^{*}/N_\text {cells})} \end{aligned}$$

As an infection progresses from $T(0)=N_\text {cells}$ where $R(t=0)=R_0$, eventually the number of uninfected cells reaches a critical value, $T(t) = T^{*}$, such that

49 $$\begin{aligned}&R(t) = 1 \equiv \frac{p\tau _I \cdot \gamma }{1+c/(\beta T^{*}/s)}&\frac{T^{*}}{N_\text {cells}} = \frac{c/(\beta N_\text {cells}/s)}{\gamma p\tau _I-1} \ . \end{aligned}$$

When the number of uninfected cells remaining *T*(*t*) equals $T^{*}$, then by definition $R(t)=1$ marking the infectious cell population peak, as shown in Fig. 17(A). Thereafter, the infectious cell population declines and the number of cells that remain uninfected approaches its final value, $T(\infty )$. When the initial inoculum consists of one or a few infectious virions, the fraction of cells consumed by infections in the MFM or by established infections in the SM, $1-T(\infty )/N_\text {cells}$, can be represented by a strictly decreasing function of $T^{*}/N_\text {cells}$, Eq. (42). This makes sense because if the infection parameters are such that fewer cells need to be consumed by the infection to reach $R(t)=1$ (larger $T^{*}$), then fewer cells will have been consumed by the time the infection ends (larger $T(\infty )$), as shown in Fig. 17(B). This theoretical expression for the fraction of cells consumed is not, however, simply a function of $R_0$. Figure 17(C) shows how an infection can start with a higher $R_0$ but *R*(*t*) can decrease more rapidly as the infection progresses such that $R(t)=1$ with the same fraction of uninfected cells ($T^{*}/N_\text {cells}$), ultimately resulting in the same fraction of cells consumed, $1-T(\infty )/N_\text {cells}$.

### Generalizing impact of antiviral reducing $\gamma$ versus one reducing $\beta$ or *p*

#### Impact on the establishment probability

$\gamma$ **vs** $\beta$ The establishment probability, or $1-(\text {extinction probability})$ (Eq. (8)), for an infection initiated with a single infectious virion is given by

50 $$\begin{aligned} {\mathcal {P}}_{V \rightarrow \, \text {Establishment}}= {\mathcal {P}}_{V \rightarrow \, I }- {\mathcal {P}}_{V \rightarrow \, I }\left[ \frac{{\mathcal {B}} \cdot {\mathcal {P}}_{V \rightarrow \, \text {Establishment}}}{n_I}+1\right] ^{-n_I} \end{aligned}$$

While an antiviral reducing $\beta$ decreases both the numerator and denominator of ${\mathcal {P}}_{V \rightarrow \, I }$ (Eq. (5)), one reducing $\gamma$ decreases only the numerator. Therefore, at equal efficacy, an antiviral reducing $\gamma$ decreases ${\mathcal {P}}_{V \rightarrow \, I }$ more than one reducing $\beta$. If ${\mathcal {P}}_{V \rightarrow \, I }$ decreases monotonically as ${\mathcal {P}}_{V \rightarrow \, \text {Establishment}}$ decreases, an antiviral causing a greater decrease in ${\mathcal {P}}_{V \rightarrow \, I }$ will cause an equal or greater decrease in ${\mathcal {P}}_{V \rightarrow \, \text {Establishment}}$.

Let us show that ${\mathcal {P}}_{V \rightarrow \, I }$ is a monotonically increasing function of ${\mathcal {P}}_{V \rightarrow \, \text {Establishment}}$. Eq. (50) can be rewritten as

51 $$\begin{aligned} {\mathcal {P}}_{V \rightarrow \, I }= \frac{{\mathcal {P}}_{V \rightarrow \, \text {Establishment}}}{1-\left[ \frac{{\mathcal {B}} \cdot {\mathcal {P}}_{V \rightarrow \, \text {Establishment}}}{n_I}+1\right] ^{-n_I}} = \frac{x}{1-\left[ \frac{{\mathcal {B}} \cdot x}{n_I}+1\right] ^{-n_I}} \end{aligned}$$

where we define $x \equiv {\mathcal {P}}_{V \rightarrow \, \text {Establishment}}$ for convenience. Taking the derivative of the above with respect to *x* yields

52 $$\begin{aligned} \frac{\partial {\mathcal {P}}_{V \rightarrow \, I }}{\partial x}&= \frac{\partial }{\partial x} \left( \frac{x}{1-\left[ \frac{{\mathcal {B}} \cdot x}{n_I}+1\right] ^{-n_I}} \right) \nonumber \\&= \frac{\left( 1-\left[ \frac{{\mathcal {B}} \cdot x}{n_I}+1\right] ^{-n_I}\right) \cdot \frac{\partial }{\partial x}(x)-x\cdot \frac{\partial }{\partial x}\left( 1-\left[ \frac{{\mathcal {B}} \cdot x}{n_I}+1\right] ^{-n_I}\right) }{\left( 1-\left[ \frac{{\mathcal {B}} \cdot x}{n_I}+1\right] ^{-n_I}\right) ^2} \nonumber \\&= \frac{1-\left[ \frac{{\mathcal {B}} \cdot x}{n_I}+1\right] ^{-n_I}-{\mathcal {B}} \cdot x\left[ \frac{{\mathcal {B}} \cdot x}{n_I}+1\right] ^{-n_I-1}}{\left( 1-\left[ \frac{{\mathcal {B}} \cdot x}{n_I}+1\right] ^{-n_I}\right) ^2} \nonumber \\&= \frac{\left[ \frac{{\mathcal {B}} \cdot x}{n_I}+1\right] ^{-n_I-1}\left( \left[ \frac{{\mathcal {B}} \cdot x}{n_I}+1\right] ^{n_I+1}-\left[ \frac{{\mathcal {B}} \cdot x}{n_I}+1\right] -{\mathcal {B}} \cdot x \right) }{\left( 1-\left[ \frac{{\mathcal {B}} \cdot x}{n_I}+1\right] ^{-n_I}\right) ^2} \nonumber \\&= \frac{\left[ \frac{{\mathcal {B}} \cdot x}{n_I}+1\right] ^{n_I+1}-\left[ \frac{{\mathcal {B}} \cdot x}{n_I}+1\right] -{\mathcal {B}} \cdot x}{\left[ \frac{{\mathcal {B}} \cdot x}{n_I}+1\right] ^{n_I+1}\left( 1-\left[ \frac{{\mathcal {B}} \cdot x}{n_I}+1\right] ^{-n_I}\right) ^2} \nonumber \\ \frac{\partial {\mathcal {P}}_{V \rightarrow \, I }}{\partial x}&= \frac{\left[ \frac{{\mathcal {B}} \cdot x}{n_I}+1\right] \left[ \frac{{\mathcal {B}} \cdot x}{n_I}+1\right] ^{n_I}-\left[ \frac{{\mathcal {B}} \cdot x}{n_I}+1\right] -{\mathcal {B}} \cdot x}{\left[ \frac{{\mathcal {B}} \cdot x}{n_I}+1\right] ^{n_I+1}\left( 1-\left[ \frac{{\mathcal {B}} \cdot x}{n_I}+1\right] ^{-n_I}\right) ^2} \ . \end{aligned}$$

Using the binomial theorem, Eq. (52) can be written as

$$\begin{aligned} \frac{\partial {\mathcal {P}}_{V \rightarrow \, I }}{\partial x}&= \frac{\left[ \frac{{\mathcal {B}} \cdot x}{n_I}+1\right] \sum _{k=0}^{n_I} {n_I \atopwithdelims ()k}\left[ \frac{{\mathcal {B}} \cdot x}{n_I}\right] ^k-\left[ \frac{{\mathcal {B}} \cdot x}{n_I}+1\right] -{\mathcal {B}} \cdot x}{\left[ \frac{{\mathcal {B}} \cdot x}{n_I}+1\right] ^{n_I+1}\left( 1-\left[ \frac{{\mathcal {B}} \cdot x}{n_I}+1\right] ^{-n_I}\right) ^2} \end{aligned}$$

Since $n_I \ge 1$, we can expand the sum at least for $k = 0$ and $k = 1$, namely,

53 $$\begin{aligned} \frac{\partial {\mathcal {P}}_{V \rightarrow \, I }}{\partial x}&= \frac{\left[ \frac{{\mathcal {B}} \cdot x}{n_I}+1\right] {n_I \atopwithdelims ()0}\left[ \frac{{\mathcal {B}} \cdot x}{n_I}\right] ^0+\left[ \frac{{\mathcal {B}} \cdot x}{n_I}+1\right] {n_I \atopwithdelims ()1}\left[ \frac{{\mathcal {B}} \cdot x}{n_I}\right] ^1+\left[ \frac{{\mathcal {B}} \cdot x}{n_I}+1\right] \sum _{k=2}^{n_I} {n_I \atopwithdelims ()k}\left[ \frac{{\mathcal {B}} \cdot x}{n_I}\right] ^k-\left[ \frac{{\mathcal {B}} \cdot x}{n_I}+1\right] -{\mathcal {B}} \cdot x}{\left[ \frac{{\mathcal {B}} \cdot x}{n_I}+1\right] ^{n_I+1}\left( 1-\left[ \frac{{\mathcal {B}} \cdot x}{n_I}+1\right] ^{-n_I}\right) ^2} \nonumber \\&= \frac{\left[ \frac{{\mathcal {B}} \cdot x}{n_I}+1\right] +{\mathcal {B}} \cdot x\left[ \frac{{\mathcal {B}} \cdot x}{n_I}+1\right] +\left[ \frac{{\mathcal {B}} \cdot x}{n_I}+1\right] \sum _{k=2}^{n_I} {n_I \atopwithdelims ()k}\left[ \frac{{\mathcal {B}} \cdot x}{n_I}\right] ^k-\left[ \frac{{\mathcal {B}} \cdot x}{n_I}+1\right] -{\mathcal {B}} \cdot x}{\left[ \frac{{\mathcal {B}} \cdot x}{n_I}+1\right] ^{n_I+1}\left( 1-\left[ \frac{{\mathcal {B}} \cdot x}{n_I}+1\right] ^{-n_I}\right) ^2} \nonumber \\ \frac{\partial {\mathcal {P}}_{V \rightarrow \, I }}{\partial x}&= \frac{\frac{({\mathcal {B}} \cdot x)^2}{n_I}+\left[ \frac{{\mathcal {B}} \cdot x}{n_I}+1\right] \sum _{k=2}^{n_I} {n_I \atopwithdelims ()k}\left[ \frac{{\mathcal {B}} \cdot x}{n_I}\right] ^k}{\left[ \frac{{\mathcal {B}} \cdot x}{n_I}+1\right] ^{n_I+1}\left( 1-\left[ \frac{{\mathcal {B}} \cdot x}{n_I}+1\right] ^{-n_I}\right) ^2} \ . \end{aligned}$$

Since $({\mathcal {B}} \cdot x/n_I)+1 \in [1,{\mathcal {B}} ]$, $\frac{\partial {\mathcal {P}}_{V \rightarrow \, I }}{\partial x} = \frac{\partial {\mathcal {P}}_{V \rightarrow \, I }}{\partial {\mathcal {P}}_{V \rightarrow \, \text {Establishment}}} \ge 0$ $\forall (x,{\mathcal {B}},n_I)$. This means that ${\mathcal {P}}_{V \rightarrow \, I }$ is a monotonically increasing function of ${\mathcal {P}}_{V \rightarrow \, \text {Establishment}}$. Since an antiviral reducing $\gamma$ causes a greater decrease in ${\mathcal {P}}_{V \rightarrow \, I }$ than one reducing $\beta$, at equal efficacy an antiviral reducing $\gamma$ will decrease ${\mathcal {P}}_{V \rightarrow \, \text {Establishment}}$ *at least as much or more so* than one reducing $\beta$.

$\gamma$ **vs** *p* The establishment probability, or $1-(\text {extinction probability})$ (Eq. (8)), for an infection initiated with a single infectious virion in the presence of an antiviral reducing $\gamma$ is given by

54 $$\begin{aligned} {\mathcal {P}}_{V \rightarrow \, \text {Establishment}}^{(\gamma )} = (1-\varepsilon ){\mathcal {P}}_{V \rightarrow \, I }- (1-\varepsilon ) {\mathcal {P}}_{V \rightarrow \, I }\left[ \frac{{\mathcal {B}} \cdot {\mathcal {P}}_{V \rightarrow \, \text {Establishment}}^{(\gamma )}}{n_I}+1\right] ^{-n_I} \ , \end{aligned}$$

because $\gamma$ is in the numerator of ${\mathcal {P}}_{V \rightarrow \, I }$, whereas an antiviral reducing *p* is given by

55 $$\begin{aligned} {\mathcal {P}}_{V \rightarrow \, \text {Establishment}}^{(p)} = {\mathcal {P}}_{V \rightarrow \, I }- {\mathcal {P}}_{V \rightarrow \, I }\left[ \frac{(1-\varepsilon ){\mathcal {B}} \cdot {\mathcal {P}}_{V \rightarrow \, \text {Establishment}}^{(p)}}{n_I}+1\right] ^{-n_I} \ , \end{aligned}$$

because *p* is in ${\mathcal {B}} =p\tau _I$ but does not appear in ${\mathcal {P}}_{V \rightarrow \, I }$, such that

56 $$\begin{aligned} {\mathcal {P}}_{V \rightarrow \, \text {Establishment}}^{(\gamma )} = (1-\varepsilon )\ {\mathcal {P}}_{V \rightarrow \, \text {Establishment}}^{(p)} \ . \end{aligned}$$

Since $\varepsilon \in (0,1]$, an antiviral reducing $\gamma$ will always decrease the establishment probability (${\mathcal {P}}_{V \rightarrow \, \text {Establishment}}$) *at least as much or more so* than an antiviral reducing *p*.

#### Impact on the fraction of cells consumed by established infections

The median fraction of cells consumed by established infections, Eq. (40), decreases monotonically as you decrease the argument of $W_0$ by reducing either the term $[\gamma (\beta /s)/c \cdot V(0)]$ or the term $N_\text {cells}/T^{*}= (\beta /s)/c\cdot [\gamma p \tau _I - 1]$ or both.

$\gamma$ versus $\beta$ At a given efficacy, an antiviral reducing $\gamma$ or $\beta$ decreases the first term by the same amount, but one reducing $\gamma$ will decreases $N_\text {cells}/T^{*}$ more than one reducing $\beta$. Therefore, an antiviral reducing $\gamma$ will always decrease the fraction of cells consumed *at least as much or more so* than an antiviral reducing $\beta$.

$\gamma$ versus *p* At a given efficacy, an antiviral reducing $\gamma$ or *p* decreases $N_\text {cells}/T^{*}$ by the same amount, but one reducing $\gamma$ will also reduce the first term whereas one reducing *p* has no effect on the first term. Therefore, an antiviral reducing $\gamma$ will always decrease the fraction of cells consumed *at least as much or more so* than an antiviral reducing *p*.

### Supplementary Information

Supplementary Information.

## Data Availability

There are no primary data in the paper. The code for the mean-field model (MFM) and the stochastic model (SM) is freely available on GitHub (https://github.com/cquir/vir-inf-stoch).
