# Supplementary material for: The effect of random virus failure following cell entry on infection outcome and the success of antiviral therapy
Source: Sci Rep. 2023 Oct 11;13:17243. doi: 10.1038/s41598-023-44180-w (PMC10567758; doi:10.1038/s41598-023-44180-w)
Supplement: Supplementary file 1 — Supplementary Information. [file 41598_2023_44180_MOESM1_ESM.pdf]

Supplementary Material for

The effect of random virus failure following cell entry on infection outcome and the success of antiviral therapy

Christian Quirouette[1], Daniel Cresta[1], Jizhou Li[2], Kathleen P. Wilkie[3], Haozhao Liang[4,¤], Catherine A.A. Beauchemin[1,2,*]

[1] Department of Physics, Toronto Metropolitan University, Toronto, Canada

[2] Interdisciplinary Theoretical and Mathematical Sciences (iTHEMS), RIKEN, Wako, Japan

[3] Department of Mathematics, Toronto Metropolitan University, Toronto, Canada

[4] Nishina Center for Accelerator-Based Science (RNC), RIKEN, Wako, Japan

¤Current address: Department of Physics, University of Tokyo, Tokyo, Japan

[*] Corresponding author: cbeau@torontomu.ca

August 10, 2023

S1 MFM with higher order moments

The goal of this section is to use a simpler version of our SM to derive a MFM which will include higher order moment terms, and to compare the MFM solution with and without the higher order moment terms.

Let us express a simpler version of our SM as transitions, namely,

$$
\begin{aligned}
V + T &\xrightarrow{\gamma\beta/s} E \\
V + T &\xrightarrow{(1-\gamma)\beta/s} T \\
E &\xrightarrow{1/\tau_E} I \\
I &\xrightarrow{1/\tau_I} \varnothing \\
I &\xrightarrow{p} I + V \\
V &\xrightarrow{c} \varnothing
\end{aligned}
\tag{S1}
$$

Important differences with our more complex SM (Eqn. (1)) are that, for the simpler SM, the number of each transition occurring over a time step is Poisson distributed, there is a single compartment for the eclipse and infectious phase, and one successful virion is assumed to cause the infection of one cell.

Let $q_{i,j,k,l}(t)$ represent the probability that $[T(t), E(t), I(t), V(t)] = [i, j, k, l]$. Following a similar procedure as outlined in [1], assuming the time step Δt is sufficiently small that the probability of one transition occurring over Δt is approximately given by the rate of the respective Poisson distribution, and the probability of more than one transition occurring over Δt is negligible, we have

$$
\begin{aligned}
&q_{i,j,k,l}(t+\Delta t) \\
&= \Delta t \cdot \gamma\beta/s \cdot (i+1)(l+1) \cdot q_{i+1,j-1,k,l+1}(t) + \Delta t \cdot (1-\gamma)\beta/s \cdot i(l+1) \cdot q_{i,j,k,l+1}(t) \\
&\quad + \Delta t \cdot 1/\tau_E \cdot (j+1) \cdot q_{i,j+1,k-1,l}(t) + \Delta t \cdot 1/\tau_I \cdot (k+1) \cdot q_{i,j,k+1,l}(t) \\
&\quad + \Delta t \cdot p \cdot k \cdot q_{i,j,k,l-1}(t) + \Delta t \cdot c \cdot (l+1) q_{i,j,k,l+1}(t) \\
&\quad + \left[1 - \Delta t \cdot (p \cdot k + c \cdot l + 1/\tau_E \cdot j + 1/\tau_I \cdot k + \beta/s \cdot il)\right] q_{i,j,k,l}(t)
\end{aligned}
\tag{S2}
$$

Subtracting $q_{i,j,k,l}(t)$ from Eqn. (S2), then dividing by Δt, and finally letting $\Delta t \to 0$, yields the forward Kolmogorov differential equation,

$$\begin{aligned}\frac{\mathrm{d}q_{i,j,k,l}}{\mathrm{d}t} =& \gamma\beta/s \cdot (i+1)(l+1) \cdot q_{i+1,j-1,k,l+1} + (1-\gamma)\beta/s \cdot i(l+1) \cdot q_{i,j,k,l+1} \\ &+ 1/\tau_E \cdot (j+1) \cdot q_{i,j+1,k-1,l} + 1/\tau_I \cdot (k+1) \cdot q_{i,j,k+1,l} \\ &+ p \cdot k \cdot q_{i,j,k,l-1} + c \cdot (l+1) \cdot q_{i,j,k,l+1} \\ &- (p \cdot k + c \cdot l + 1/\tau_E \cdot j + 1/\tau_I \cdot k + \beta/s \cdot il) \cdot q_{i,j,k,l}\end{aligned} \tag{S3}$$

Multiplying Eqn. (S3) by $\mathrm{e}^{\theta i+\phi j+\psi k+\zeta l}$ and summing over (i,j,k,l), Eqn. (S3) becomes

$$\begin{aligned}&\frac{\mathrm{d}}{\mathrm{d}t}\sum_{i,j,k,l} q_{i,j,k,l} \cdot \mathrm{e}^{\theta i+\phi j+\psi k+\zeta l} \\ &= \gamma\beta/s \sum_{i,j,k,l} (i+1)(l+1) \cdot q_{i+1,j-1,k,l+1} \cdot \mathrm{e}^{\theta i+\phi j+\psi k+\zeta l} \\ &\quad + (1-\gamma)\beta/s \sum_{i,j,k,l} i(l+1) \cdot q_{i,j,k,l+1} \cdot \mathrm{e}^{\theta i+\phi j+\psi k+\zeta l} \\ &\quad + 1/\tau_E \sum_{i,j,k,l} (j+1) \cdot q_{i,j+1,k-1,l} \cdot \mathrm{e}^{\theta i+\phi j+\psi k+\zeta l} \\ &\quad + 1/\tau_I \sum_{i,j,k,l} (k+1) \cdot q_{i,j,k+1,l} \cdot \mathrm{e}^{\theta i+\phi j+\psi k+\zeta l} \\ &\quad + p \sum_{i,j,k,l} k \cdot q_{i,j,k,l-1} \cdot \mathrm{e}^{\theta i+\phi j+\psi k+\zeta l} \\ &\quad + c \sum_{i,j,k,l} (l+1) \cdot q_{i,j,k,l+1} \cdot \mathrm{e}^{\theta i+\phi j+\psi k+\zeta l} \\ &\quad - \sum_{i,j,k,l} (p \cdot k + c \cdot l + 1/\tau_E \cdot j + 1/\tau_I \cdot k + \beta/s \cdot il) \cdot q_{i,j,k,l} \cdot \mathrm{e}^{\theta i+\phi j+\psi k+\zeta l}\end{aligned} \tag{S4}$$

Substituting the moment generating function $M = \sum_{i,j,k,l} q_{i,j,k,l} \cdot \mathrm{e}^{\theta i+\phi j+\psi k+\zeta l}$ into Eqn. (S4), one can obtain

$$\begin{aligned}\frac{\partial M}{\partial t} =& \beta/s\left[\gamma \mathrm{e}^{\phi-\theta-\zeta} + (1-\gamma)\mathrm{e}^{-\zeta} - 1\right]\frac{\partial^2 M}{\partial\theta\partial\zeta} \\ &+ (1/\tau_E)\left[\mathrm{e}^{\psi-\phi} - 1\right]\frac{\partial M}{\partial\phi} + (1/\tau_I)\left[\mathrm{e}^{-\psi} - 1\right]\frac{\partial M}{\partial\psi} \\ &+ p\left[\mathrm{e}^{\zeta} - 1\right]\frac{\partial M}{\partial\psi} + c\left[\mathrm{e}^{-\zeta} - 1\right]\frac{\partial M}{\partial\zeta}\end{aligned} \tag{S5}$$

Eqn. (S5) can be used to derive a MFM. For example, to derive the mean-field equation for the target cell population (T), one can use the following property of the moment generating function,

$$\frac{\mathrm{dE}[T]}{\mathrm{d}t} = \frac{\partial}{\partial t}\frac{\partial M}{\partial\theta}\bigg|_{\theta=\phi=\psi=\zeta=0} \tag{S6}$$

The MFM one can derive from Eqn. (S5) is given by

$$
\begin{aligned}
\frac{\mathrm{dE}[T]}{\mathrm{d}t} &= -\gamma\beta/s \cdot \mathrm{E}[TV] \\
\frac{\mathrm{dE}[E]}{\mathrm{d}t} &= \gamma\beta/s \cdot \mathrm{E}[TV] - 1/\tau_E \cdot \mathrm{E}[E] \\
\frac{\mathrm{dE}[I]}{\mathrm{d}t} &= 1/\tau_E \cdot \mathrm{E}[E] - 1/\tau_I \cdot \mathrm{E}[I] \\
\frac{\mathrm{dE}[V]}{\mathrm{d}t} &= p \cdot \mathrm{E}[I] - c \cdot \mathrm{E}[V] - \beta/s \cdot \mathrm{E}[TV] \\
\frac{\mathrm{dE}[TT]}{\mathrm{d}t} &= \gamma\beta/s \cdot \mathrm{E}[TV] - 2\gamma\beta/s \cdot \mathrm{E}[TTV] \\
\frac{\mathrm{dE}[TE]}{\mathrm{d}t} &= -\gamma\beta/s \cdot \mathrm{E}[TEV] - \gamma\beta/s \cdot \mathrm{E}[TV] + \gamma\beta/s \cdot \mathrm{E}[TTV] - 1/\tau_E \cdot \mathrm{E}[TE] \\
\frac{\mathrm{dE}[TI]}{\mathrm{d}t} &= -\gamma\beta/s \cdot \mathrm{E}[TIV] + (1/\tau_E) \cdot \mathrm{E}[TE] - (1/\tau_I) \cdot \mathrm{E}[TI] \\
\frac{\mathrm{dE}[TV]}{\mathrm{d}t} &= -\gamma\beta/s \cdot \mathrm{E}[TVV] + \gamma\beta/s \cdot \mathrm{E}[TV] + p \cdot \mathrm{E}[TI] - c \cdot \mathrm{E}[TV] - \beta/s \cdot \mathrm{E}[TTV] \\
\frac{\mathrm{dE}[EE]}{\mathrm{d}t} &= \gamma\beta/s \cdot \mathrm{E}[TV] + 2\gamma\beta/s \cdot \mathrm{E}[TEV] - 1/\tau_E \cdot \mathrm{E}[E] - 2(1/\tau_E) \cdot \mathrm{E}[EE] \\
\frac{\mathrm{dE}[EI]}{\mathrm{d}t} &= \gamma\beta/s \cdot \mathrm{E}[TIV] - 1/\tau_E \cdot \mathrm{E}[EI] - 1/\tau_E \cdot \mathrm{E}[E] + 1/\tau_E \cdot \mathrm{E}[EE] - 1/\tau_I \cdot \mathrm{E}[EI] \\
\frac{\mathrm{dE}[EV]}{\mathrm{d}t} &= \gamma\beta/s \cdot \mathrm{E}[TVV] - \gamma\beta/s \cdot \mathrm{E}[TV] - 1/\tau_E \cdot \mathrm{E}[EV] + p \cdot \mathrm{E}[EI] - c \cdot \mathrm{E}[EV] \\
&\quad - \beta/s \cdot \mathrm{E}[TEV] \\
\frac{\mathrm{dE}[II]}{\mathrm{d}t} &= 1/\tau_E \cdot \mathrm{E}[E] + 2(1/\tau_E) \cdot \mathrm{E}[EI] + 1/\tau_I \cdot \mathrm{E}[I] - 2(1/\tau_I) \cdot \mathrm{E}[II] \\
\frac{\mathrm{dE}[IV]}{\mathrm{d}t} &= 1/\tau_E \cdot \mathrm{E}[E] - 1/\tau_E \cdot \mathrm{E}[IV] + p \cdot \mathrm{E}[II] - c \cdot \mathrm{E}[IV] - \beta/s \cdot \mathrm{E}[TIV] \\
\frac{\mathrm{dE}[VV]}{\mathrm{d}t} &= p \cdot \mathrm{E}[I] + 2p \cdot \mathrm{E}[IV] + c \cdot \mathrm{E}[V] - 2c \cdot \mathrm{E}[VV] + \beta/s \cdot \mathrm{E}[TV] - \beta/s \cdot \mathrm{E}[TVV]
\end{aligned} \tag{S7}
$$

where the following equation can be used to approximate the third order moment terms,

$$
\begin{aligned}
\mathrm{E}[ABC] \approx\, & \mathrm{E}[A](\mathrm{E}[BC] - \mathrm{E}[B]\mathrm{E}[C]) + \mathrm{E}[B](\mathrm{E}[AC] - \mathrm{E}[A]\mathrm{E}[C]) \\
& + \mathrm{E}[C](\mathrm{E}[AB] - \mathrm{E}[A]\mathrm{E}[B]) - \mathrm{E}[A]\mathrm{E}[B]\mathrm{E}[C]
\end{aligned} \tag{S8}
$$

If we assume the covariance $\mathrm{cov}(T, V) = 0$ then $\mathrm{E}[TV] = \mathrm{E}[T]\mathrm{E}[V]$, and we obtain a simpler system of differential equations without second order moment terms, namely,

$$
\begin{aligned}
\frac{\mathrm{dE}[T]}{\mathrm{d}t} &= -\gamma\beta/s \cdot \mathrm{E}[T]\mathrm{E}[V] \\
\frac{\mathrm{dE}[E]}{\mathrm{d}t} &= \gamma\beta/s \cdot \mathrm{E}[T]\mathrm{E}[V] - 1/\tau_E \cdot \mathrm{E}[E] \\
\frac{\mathrm{dE}[I]}{\mathrm{d}t} &= 1/\tau_E \cdot \mathrm{E}[E] - 1/\tau_I \cdot \mathrm{E}[I] \\
\frac{\mathrm{dE}[V]}{\mathrm{d}t} &= p \cdot \mathrm{E}[I] - c \cdot \mathrm{E}[V] - \beta/s \cdot \mathrm{E}[T]\mathrm{E}[V]
\end{aligned} \tag{S9}
$$

Fig S1 shows that, for an infection with initially a single infectious virion, there is no significant disagreement between the infectious virion time course obtained from the MFM with and without second order moments, Eqn. (S7) and (S9) respectively, using a standard numerical ODE solver.

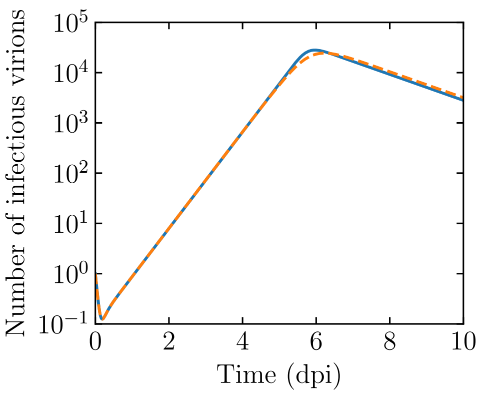

Figure S1: **MFM solution with versus without second order moments.** Infectious virion time course obtained from the MFM with second order moments (blue solid line, Eqn. (S7)) compared to that obtained from the MFM without second order moments (orange dashed line, Eqn. (S9)), for an infection with initially a single infectious virion. Infection parameters are the same as in Fig 4.

S2 Inoculum size

In the main text, the initial number of virions $V_0 = 1$ IV and we explored varying the efficacy ε. Now, we fix $\varepsilon = 0.8$ and we explore varying V_0. Fig S2(A) and Fig S2(B) shows the establishment probability or the median fraction of cells consumed by established infections, respectively, as a function of V_0 for antivirals with efficacy $\varepsilon = 0.8$ reducing β, p or γ. As V_0 increases, the establishment probability tends to 100% and differences in the establishment probability for the 3 antiviral modes of action disappear. For $V_0 \ll N_{\text{cells}} = 4 \times 10^4$ cells, the theoretical expression for the median fraction of cells consumed by the infection, Eqn. (42), is insensitive to V_0.

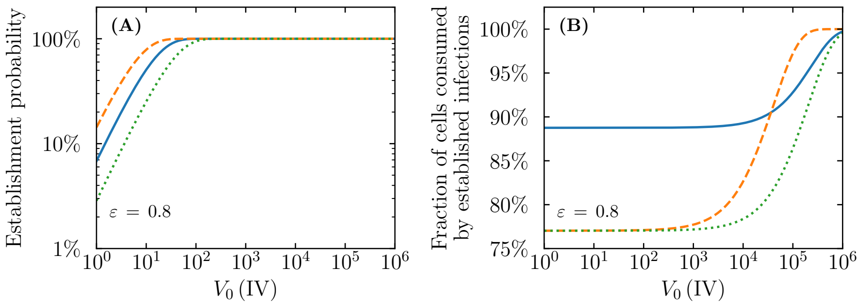

Figure S2: **Inoculum size.** (A) The establishment probability or (B) the median fraction of cells consumed by established infections as a function of the initial number of infectious virions V_0 for antivirals with efficacy $\varepsilon = 0.8$ acting either to reduce the virus entry rate, $\beta \to (1-\varepsilon)\beta$ (blue solid line), the virus production rate, $p \to (1-\varepsilon)p$ (orange dashed line), or the probability of a successful cell infection post virus entry, $\gamma \to (1-\varepsilon)\gamma$ (green dotted line). The parameters were the same as in Fig 4.

Table S1: Parameter sets used in Czuppon et al. [3].

Parameter set	p [IV/(cell · h)]	N_{cells} [cells]	R_0*	τ_I [h]	c [h^{-1}]	n_I
Lower burst size	11.2/24	4×10^4	7.69	24/0.595	10/24	1
Higher burst size	112/24	4×10^3	7.69	24/0.595	10/24	1

* $\beta = [cR_0/\tau_I]/[N_{\text{cells}}(p - R_0/\tau_I)]$

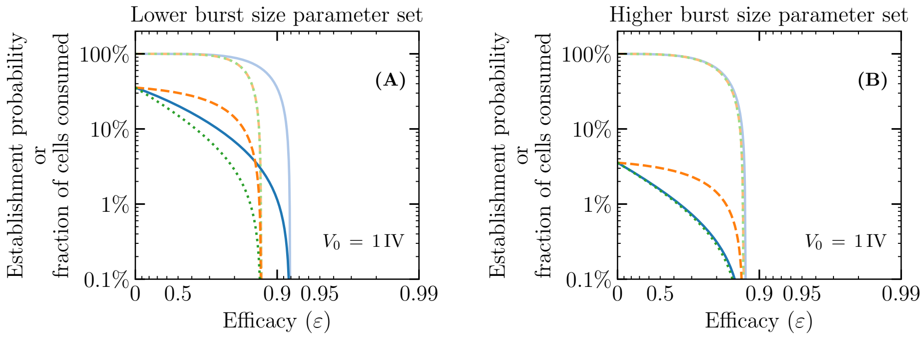

Figure S3: **Lower vs. higher burst size parameter set.** The establishment probability (dark colours) or the fraction of cells consumed by established infections (pale colours) given that there is initially only one infectious virion as a function of efficacy (ε) for antivirals acting either to reduce the virus entry rate, $\beta \to (1-\varepsilon)\beta$ (blue solid lines), the virus production rate, $p \to (1-\varepsilon)p$ (orange dashed lines), or the probability of a successful cell infection post virus entry, $\gamma \to (1-\varepsilon)\gamma$ (green dotted lines) for the parameter set with (A) the lower burst size or (B) the higher burst size (see Table S1) where our additional infection parameters were set to $\gamma = 1$ cell/IV and $s = 1$ mL.

S3 Parameter set with a higher burst size

Here, we investigate the other parameter set explored in Czuppon et al. [3]. The difference between the two parameter sets is a 10-fold decrease of the number of cells N_{cells}, a 10-fold increase of the virus production rate p (hence, of the average burst size $\mathcal{B} = p\tau_I$) and a corresponding ∼10-fold decrease of βN_{cells} (see Table S1).

Fig S3 shows both the establishment probability and the median fraction of cells consumed by established infections given that there is initially only one infectious virion as a function of antiviral efficacy (ε) for antivirals reducing β, p or γ for the parameter set with (A) the lower burst size ($\mathcal{B} = 18.8$ IV/cell) or (B) the higher burst size ($\mathcal{B} = 188$ IV/cell).

For the parameter set with the higher burst size, an antiviral reducing γ or β have a similar effect on the establishment probability, better than that for an antiviral reducing p. Due to the higher burst size, the establishment probability is approximately given by the probability that the initial infectious virion causes a productive cell infection, $\mathcal{P}_{V \to \text{Establishment}} = \mathcal{P}_{V \to I} - 1/\mathcal{B} \approx \mathcal{P}_{V \to I}$ for $n_I = 1$ (see Methods for more details). The probability that the initial infectious virion will cause a productive cell infection is given by the ratio between the rate of successful cell infection per infectious virion and the rate of virion loss $(\gamma\beta N_{\text{cells}}/s)/(c+\beta N_{\text{cells}}/s)$. Since the rate of virion entry into cells is much lower than the rate of virion loss of infectivity ($\beta N_{\text{cells}}/s \ll c$) the rate of virion loss is mostly governed by the rate of virion loss of infectivity ($(c + \beta N_{\text{cells}}/s) \approx c$). Therefore, the establishment probability is approximately given by $(\gamma\beta N_{\text{cells}}/s)/c$ which is affected by γ or β the same but not affected by p.

For the parameter set with the higher burst size, an antiviral reducing either β, γ or p results in the same median fraction of cells consumed by established infections at equal efficacy. This is because, for burst

size ($\mathcal{B} = p\tau_I$) sufficiently large that $\gamma p\tau_I - 1 \approx \gamma p\tau_I$, we have $T^*/N_{\text{cells}} = [c/(\beta N_{\text{cells}}/s)]/[\gamma p\tau_I - 1] \approx [c/(\beta N_{\text{cells}}/s)]/[\gamma p\tau_I]$. As such, an antiviral acting on any of β, p or γ at the same efficacy will reduce the median fraction of cells infected by established infections (a monotonically decreasing fraction of T^*/N_{cells}) by the same amount.

S4 Post-exposure antiviral therapy

In the main text, we have considered pre-exposure antiviral therapy for an infection initiated with a number of infectious virions. Now, as Czuppon et al. [3] have done, let us also explore antiviral therapy for an infection initiated with only one infectious cell. This may be representative of post-exposure antiviral therapy as it is possible that by the time an antiviral has been given after exposure, the virus has had time to cause some infectious cells.

With $n_I = 1$, the establishment probability given that there is initially one infectious cell (see Methods for derivation) is given by

$$\mathcal{P}_{I\to\text{ Establishment}} = 1 - \frac{1}{R_0} = 1 - \frac{c + \beta N_{\text{cells}}/s}{p\tau_I \cdot \gamma\beta N_{\text{cells}}/s} = 1 - \frac{1 + c/(\beta N_{\text{cells}}/s)}{\gamma \cdot p\tau_I} \tag{S10}$$

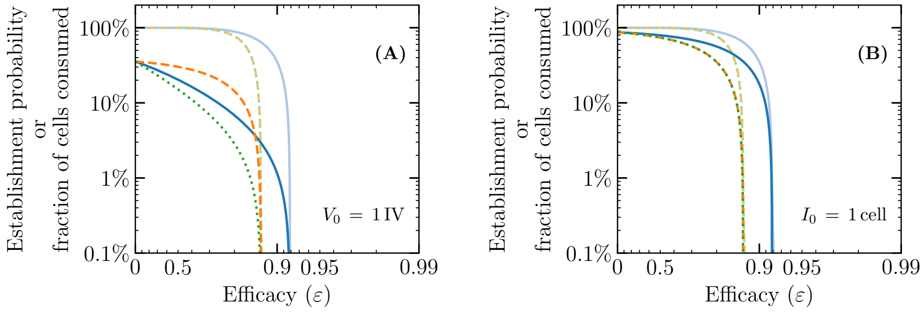

Figure S4: **Pre-exposure vs. post-exposure antiviral therapy.** The establishment probability (dark colours) or the median fraction of cells consumed by established infections (pale colours) given that there is initially only (A) one infectious virion or (B) one infectious cell as a function of efficacy (ε) for antivirals acting either to reduce the virus entry rate, $\beta \to (1-\varepsilon)\beta$ (blue solid lines), the virus production rate, $p \to (1-\varepsilon)p$ (orange dashed lines), or the probability of a successful cell infection post virus entry, $\gamma \to (1-\varepsilon)\gamma$ (green dotted lines). The parameters were the same as in Fig 4.

Fig S4 shows the establishment probability and the median fraction of cells consumed by established infections given that there is initially only (A) one infectious virion or (B) one infectious cell as a function of antiviral efficacy (ε) for antivirals reducing β, p or γ.

Like Czuppon et al. [3], with initially one infectious cell, we find that an antiviral reducing p is better than an antiviral reducing β to reduce the establishment probability. Reducing p affects only the denominator in the expression for the establishment probability given that there is initially one infectious cell (Eqn. (S10)). Whereas, reducing β affects both the numerator and denominator (Eqn. (S10)). Unlike Czuppon et al. [3], with initially one infectious cell, we find that an antiviral reducing p or γ have the same effect on the establishment probability (Eqn. (S10)).

In addition, there is no noticeable difference in the median fraction of cells consumed by established infections given that there is initially one infectious virion or one infectious cell. The initial number of infectious virions or cells is small and hence has a negligible effect on the median fraction of cells consumed by established infections (see Eqn. (42)).

S5 Infection risk reduction

Conway et al. [2] showed that, under pre-exposure HIV antiviral therapy, reverse transcriptase inhibitors (RTIs) reducing the cell infection rate β are more effective than protease inhibitors reducing the virus production rate p, at reducing the risk of infection.

In Conway et al. [2], the probability of having n infectious virions in the exposure inoculum is given by the binomial probability mass function, $\binom{N_0}{n} Q_c^n (1-Q_c)^{N_0-n}$, where N_0 is the total number of virions in the exposure inoculum and Q_c represents the fraction of virions that are infectious. The risk of infection, i.e. the likelihood of infection establishment given the exposure inoculum, is then given by

$$\text{Risk} = 1 - \sum_{n=0}^{N_0} \binom{N_0}{n} Q_c^n (1-Q_c)^{N_0-n} \cdot \mathcal{P}_{V\to\,\text{Extinction}}{}^n = 1 - [1 - Q_c \cdot (1 - \mathcal{P}_{V\to\,\text{Extinction}})]^{N_0} \tag{S11}$$

where $\mathcal{P}_{V\to\,\text{Extinction}}$ is the extinction probability given an infection initiated with only one infectious virion (denoted by q therein).

For $n_I = 1$, as in Conway et al. [2], our expression for $\mathcal{P}_{V\to\,\text{Extinction}}$ reduces to

$$\mathcal{P}_{V\to\,\text{Extinction}} = (1 - \mathcal{P}_{V\to\,I}) + \frac{1}{\mathcal{B}} = 1 - \frac{\gamma}{1 + c/(\beta N_\text{cells}/s)} - \frac{1}{p\tau_I}\ . \tag{S12}$$

In this case, the difference between our expression for the extinction probability and theirs, is the inclusion of parameter γ. RTIs prevent the transcription of viral DNA from viral RNA, a necessary replication step post virus entry. One could then argue that an antiviral reducing γ better captures the mode of action of a RTI. With the addition of parameter γ in $\mathcal{P}_{V\to\,\text{Extinction}}$, we can then test, under pre-exposure HIV antiviral therapy, if a RTI represented as reducing γ is more effective than one reducing β at reducing the risk of infection.

In Conway et al. [2], all parameters are fixed except N_0 which is assumed to be uniformly distributed over the interval $[0, N_\text{max}]$, where N_max represents the maximum inoculum size, and βN_cells over a log-normal distribution $f(\beta N_\text{cells})$. The parameters of the log-normal distribution $f(\beta N_\text{cells})$ are estimated from R_0 data from Ribeiro et al. [4] using the expression $\beta N_\text{cells} = (cR_0/\tau_I)/(p - 1/\tau_I)$. The maximum inoculum size N_max is then determined such that, without antivirals, the risk of infection averaged over N_0 and βN_cells, is ∼0.3%. For sake of simplicity, herein, we fixed $\beta N_\text{cells} = (cR_0/\tau_I)/(p - 1/\tau_I)$, where R_0 is equal to the median value of 8.04 in Ribeiro et al. [4], and N_0 such that, without antivirals, the risk of infection ∼0.3%. Other infection parameters were taken from Conway et al. [2], i.e. $n_I = 1$, $\tau_I = 24$ h, $c = 23/24\ \text{h}^{-1}$, $Q_c = 10^{-3}$, and our additional infection parameters were set to $\gamma = 1$ cell/IV and $s = 1$ mL.

Fig S5(A–C) shows the infection risk reduction as a function of antiviral efficacy for pre-exposure antivirals reducing β or γ, for different values of the infectious virion production rate p explored in Conway et al. [2]. Fig S5(D–F) shows the infection risk reduction in (A–C) for an antiviral reducing γ minus that for an antiviral reducing β. For some of the parameters sets explored, under pre-exposure antiviral therapy, a RTI represented as reducing γ is shown to be better than one reducing β, at reducing the risk of infection.

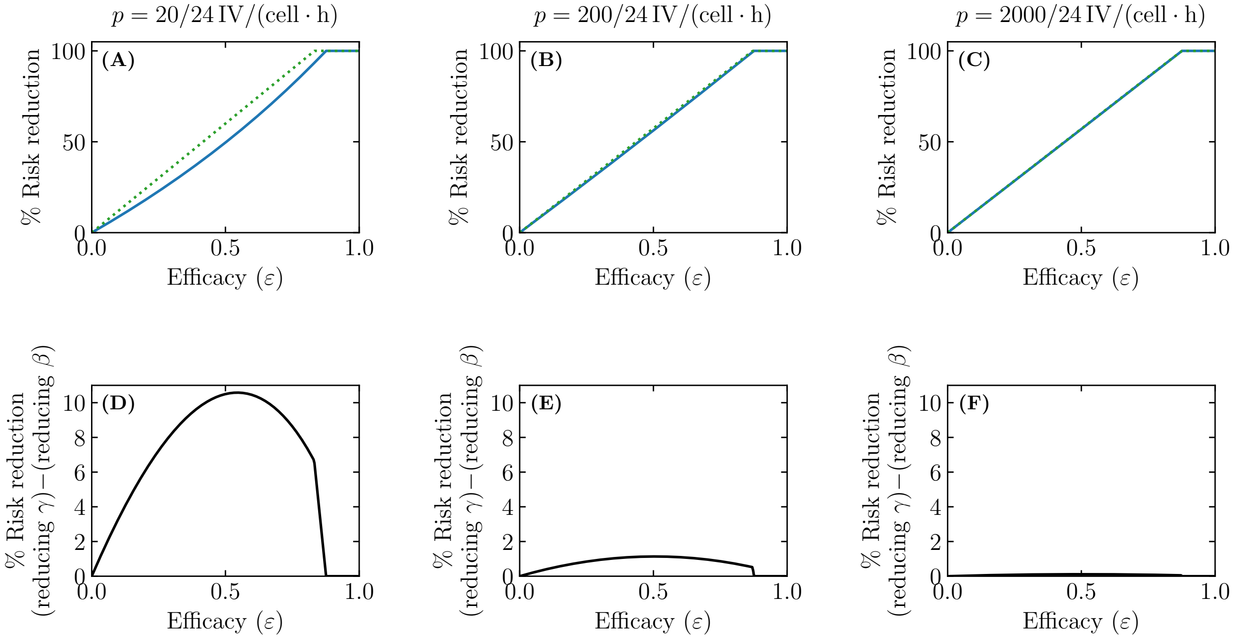

Figure S5: **Infection risk reduction.** (A–C) Infection risk reduction as a function of efficacy (ε) for antivirals acting either to reduce the virus entry rate, $\beta \to (1-\varepsilon)\beta$ (blue solid line), or the probability of a successful cell infection post virus entry, $\gamma \to (1-\varepsilon)\gamma$ (green dotted line), for different values of the infectious virion production rate p explored. Infection risk reduction for a given efficacy $\varepsilon \neq 0$ is calculated as $100\% \cdot (\text{Risk}_{\varepsilon=0} - \text{Risk}_{\varepsilon\neq 0})/(\text{Risk}_{\varepsilon\neq 0})$. (D–F) Infection risk reduction in (A–C) for an antiviral reducing γ minus that for an antiviral reducing β.

S6 Additional figures

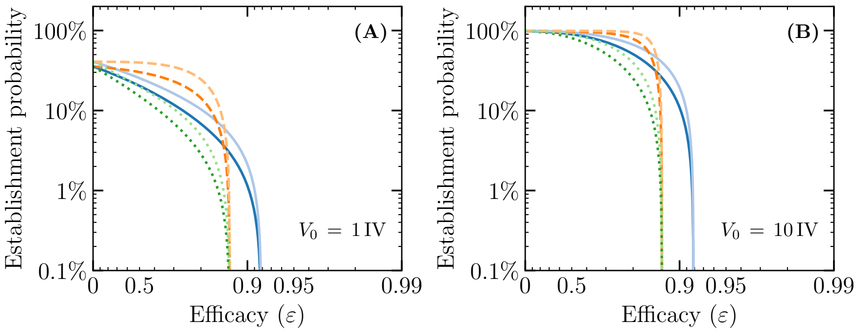

Figure S6: **Effect of infectious phase duration distribution on the ability of antivirals to reduce the establishment probability.** The establishment probability given an infection initiated with (A) 1 infectious virion or (B) 10 infectious virions as a function of efficacy (ε) for antivirals acting either to reduce the virus entry rate, $\beta \to (1-\varepsilon)\beta$ (blue solid lines), the virus production rate, $p \to (1-\varepsilon)p$ (orange dashed lines), or the probability of a successful cell infection post virus entry, $\gamma \to (1-\varepsilon)\gamma$ (green dotted lines) where either $n_I = 1$ (dark colours) or $n_I = 60$ (pale colours). Unless otherwise specified, the parameters were the same as in Fig 4.

References

[1] L. J. Allen. *An introduction to stochastic processes with applications to biology.* 2010.

[2] J. M. Conway, B. P. Konrad, and D. Coombs. Stochastic analysis of pre- and postexposure prophylaxis against HIV infection. *SIAM J. Appl. Math.*, 73(2):904–928, April 18 2013. doi:10.1137/120876800.

[3] P. Czuppon, F. Débarre, A. Gonçalves, O. Tenaillon, A. S. Perelson, J. Guedj, and F. Blanquart. Success of prophylactic antiviral therapy for SARS-CoV-2: Predicted critical efficacies and impact of different drug-specific mechanisms of action. *PLOS Comput. Biol.*, 17(3):e1008752, March 1 2021. doi:10.1371/journal.pcbi.1008752.

[4] R. M. Ribeiro, L. Qin, L. L. Chavez, D. Li, S. G. Self, and A. S. Perelson. Estimation of the initial viral growth rate and basic reproductive number during acute HIV-1 infection. *J. Virol.*, 84(12):6096–6102, June 2010. doi:10.1128/JVI.00127-10.
